# Five decades of genome evolution in the globally distributed, extensively antibiotic-resistant *Acinetobacter baumannii* global clone 1

**DOI:** 10.1099/mgen.0.000052

**Published:** 2016-02-23

**Authors:** Kathryn Holt, Johanna J. Kenyon, Mohammad Hamidian, Mark B. Schultz, Derek J. Pickard, Gordon Dougan, Ruth Hall

**Affiliations:** ^1^​ Department of Biochemistry & Molecular Biology, The University of Melbourne, Royal Parade, Parkville, Victoria, Australia; ^2^​ School of Biomedical Science, Queensland University of Technology, Brisbane, Queensland, Australia; ^3^​ School of Molecular Bioscience, The University of Sydney, Sydney, New South Wales, Australia; ^4^​ Centre for Systems Genomics, The University of Melbourne, Parkville, Victoria, Australia; ^5^​ Wellcome Sanger Trust Institute, Hinxton, Cambridge, UK

**Keywords:** *Acinetobacter baumannii*, antibiotic resistance, capsule, evolution, phylogenomics, recombination

## Abstract

The majority of *Acinetobacter baumannii* isolates that are multiply, extensively and pan-antibiotic resistant belong to two globally disseminated clones, GC1 and GC2, that were first noticed in the 1970s. Here, we investigated microevolution and phylodynamics within GC1 via analysis of 45 whole-genome sequences, including 23 sequenced for this study. The most recent common ancestor of GC1 arose around 1960 and later diverged into two phylogenetically distinct lineages. In the 1970s, the main lineage acquired the AbaR resistance island, conferring resistance to older antibiotics, via a horizontal gene transfer event. We estimate a mutation rate of ∼5 SNPs genome^− 1^ year^− 1^ and detected extensive recombination within GC1 genomes, introducing nucleotide diversity into the population at >20 times the substitution rate (the ratio of SNPs introduced by recombination compared with mutation was 22). The recombination events were non-randomly distributed in the genome and created significant diversity within loci encoding outer surface molecules (including the capsular polysaccharide, the outer core lipooligosaccharide and the outer membrane protein CarO), and spread antimicrobial resistance-conferring mutations affecting the *gyrA* and *parC* genes and insertion sequence insertions activating the *ampC* gene. Both GC1 lineages accumulated resistance to newer antibiotics through various genetic mechanisms, including the acquisition of plasmids and transposons or mutations in chromosomal genes. Our data show that GC1 has diversified into multiple successful extensively antibiotic-resistant subclones that differ in their surface structures. This has important implications for all avenues of control, including epidemiological tracking, antimicrobial therapy and vaccination.

## Data Summary

1. Sequence reads for *Acinetobacter baumannii* strains A1, A297, A388, J1, D3208, J5, WM98, J7, J10, D2, D62, D30, A83, A92, A85, 6772166, RBH3, D15, D13, G7, D81, D78 and D36 have been deposited in the European Nucleotide Archive: ERP001080 (http://www.ebi.ac.uk/ena/data/view/ERP001080). Draft genome assemblies are available under the same project accession. Individual accession numbers for each read set and genome assembly are given in Table S1 (available in the online Supplementary Material).
2. Sequence reads for *Acinetobacter baumannii* strain AB0057 were deposited separately under accession number SRR1997868 and the genome assembly is available under accession number CP001182 (http://www.ncbi.nlm.nih.gov/nuccore/CP001182).
3. Finished genome assemblies were deposited for *Acinetobacter baumannii* strain A1 under accession number CP010781 (http://www.ncbi.nlm.nih.gov/nuccore/CP010781) and for strain D36 under accession number CP012952 (http://www.ncbi.nlm.nih.gov/nuccore/CP012952).
4. Annotated sequences for novel capsule (K) loci have been deposited in GenBank: KL1a, KF483599.2 (http://www.ncbi.nlm.nih.gov/nuccore/KF483599.2); KL1b, GQ406245.5 (http://www.ncbi.nlm.nih.gov/nuccore/GQ406245.5); KL17, KC118541.2 (http://www.ncbi.nlm.nih.gov/nuccore/KC118541.2); KL20, JQ684178.2 (http://www.ncbi.nlm.nih.gov/nuccore/JQ684178.2); KL40, KP100029.1 (http://www.ncbi.nlm.nih.gov/nuccore/KP100029.1).
5. Annotated sequences for novel AbaR (antibiotic resistance genomic island) variants have been deposited in GenBank: AbaR21, KM921776 (http://www.ncbi.nlm.nih.gov/nuccore/KM921776.1); AbaR5, FJ172370 (http://www.ncbi.nlm.nih.gov/nuccore/FJ172370.5); AbaR6, GQ406245 (http://www.ncbi.nlm.nih.gov/nuccore/GQ406245); AbaR26, KC665626 (http://www.ncbi.nlm.nih.gov/nuccore/KC665626.1); AbaR7, GQ406246 (http://www.ncbi.nlm.nih.gov/nuccore/GQ406246.3); AbaR3, KC118540 (http://www.ncbi.nlm.nih.gov/nuccore/KC118540.6); AbaR8, HM590877 (http://www.ncbi.nlm.nih.gov/nuc>core/HM590877.5); AbaR23, JN409449 (http://www.ncbi.nlm.nih.gov/nuccore/JN409449.3).
6. Phylogenetic trees and the results of recombination analysis are available in FigShare: https://dx.doi.org/10.4225/49/5690B341A0FDB.

## Impact Statement

There are few opportunities to track the evolution of problem bacterial clones over an extended timeframe. Here, we dissected the fine-scale evolution of an important *Acinetobacter baumannii* clone using isolates collected over three decades. In addition to a mutation rate of ∼5 SNPs genome^− 1^ year^− 1^, we detected extensive horizontal gene transfer and homologous recombination. These microevolutionary forces combined to create a diversity of subclones within global clone GC1, which are all resistant to several antibiotics. GC1 subclones also carried different capsule loci, leading to altered surface polysaccharides. Hence, capsule-targeted vaccines are unlikely to be an effective measure for control of this multidrug-resistant clone. As one of the oldest globally distributed and persistently maintained multidrug-resistant Gram-negative clones, the evolutionary history of GC1 can provide a window into the future trajectory of more recently emerged clones, such as *Klebsiella pneumoniae* CC258 and *Escherichia coli* ST131.

## Introduction

*Acinetobacter baumannii* is one of the ESKAPE pathogens (Rice, 2008) – the six main agents of hospital-acquired antibiotic-resistant infections recognized by the Infectious Diseases Society of America. *A. baumannii* are intrinsically resistant to chloramphenicol; however, isolates resistant to a wide array of antibiotics, including sulphonamides, tetracycline, ampicillin, kanamycin, gentamicin and streptomycin, have been observed since the 1970s (Devaud *et al.*, 1982; Bergogne-Bérézin & Towner, 1996). Amplified fragment length polymorphism typing (Dijkshoorn *et al.*, 1996) and later MLST revealed that the majority of such isolates belong to one of two clones (Diancourt *et al.*, 2010), which are now globally disseminated and known as global clones GC1 and GC2 (Nigro *et al.*, 2011a). In most GC1, resistance to the older antibiotics is now known to reside in a single chromosomal locus – the AbaR resistance island (Fournier *et al.*, 2006; Post & Hall, 2009; Adams *et al.*, 2010; Krizova & Nemec, 2010; Post *et al.*, 2010, 2012; Krizova *et al.*, 2011; Nigro *et al.*, 2011b; Hamidian *et al.*, 2014c; Holt *et al.*, 2015); however, one isolate contained a different transposon at the same site, suggesting an independent history (Hamidian & Hall, 2011). Resistance to newer drugs, including fluoroquinolones, third-generation cephalosporins and carbapenems, emerged in the 1980s, associated with a wide variety of genetic mechanisms, including DNA substitutions, transposition, recombination and plasmid acquisition (Bergogne-Bérézin & Towner, 1996; Zarrilli *et al.*, 2013; Antunes *et al.*, 2014; Nigro & Hall, 2016). More recently, variation in the regions responsible for surface polysaccharides has been reported in both GC1 and GC2 (Adams *et al.*, 2008; Snitkin *et al.*, 2011; Hu *et al.*, 2013; Kenyon & Hall, 2013; Wright *et al.*, 2014; Kenyon *et al.*, 2015).

MLST has shown that *A. baumannii* is made up of distinct clones of which only GC1, GC2 and a handful of others are responsible for the majority of antibiotic-resistant human infections, indicating that *A. baumannii* lineages vary in terms of their pathogenic potential (Diancourt *et al.*, 2010; Zarrilli *et al.*, 2013; Antunes *et al.*, 2014). More recently, whole-genome comparisons yielded insights into the evolution of pathogenicity in *A. baumannii* and the genus *Acinetobacter*, uncovering wide diversity in gene content, including variation in virulence determinants (Kenyon & Hall, 2013; Sahl *et al.*, 2013; Eijkelkamp *et al.*, 2014; Kenyon *et al.*, 2014b; Touchon *et al.*, 2014; Wright *et al.*, 2014). Genomic analysis of intra-clone diversity over the period in which antibiotics have been used have been utilized to examine the microevolution of clones of multiple antibiotic-resistant bacterial pathogens, including the more recently emerged ESKAPE pathogens, such as *Escherichia coli* ST131 (Petty *et al.*, 2014) and *Klebsiella pneumoniae* ST258 (Gaiarsa *et al.*, 2015). However, genomic studies of intra-clonal variation in *A. baumannii* have been limited to small or highly localized isolate samples (Adams *et al.*, 2010; Sahl *et al.*, 2015). Here, we used genomic approaches to dissect the evolution of *A. baumannii* GC1, providing insights into the ongoing evolutionary trajectory of this important human pathogen.

## Methods

### Bacterial isolates and DNA sequencing

Genomes were carefully selected for inclusion in the study in order to ensure that the sample set was representative of the broad genomic diversity of GC1 and not driven by clusters of isolates from the same time and place. Details of bacterial isolates sequenced for this study are given in Table S1 (available in the online Supplementary Material). Details of strains with publicly available genome data used in this study, without determination of the antibiotic resistance phenotype in our laboratory, are given in Table S2. Isolates for sequencing were selected from our collection of 36 GC1 isolates from Australian hospitals to represent the maximum diversity. For groups of closely related isolates from the same hospital (e.g. with the same resistance genes and AbaR island) only the first and last to be isolated were included. Three European isolates from the UK, the Netherlands and Greece, kindly supplied by Dr Kevin Towner (Nottingham University Hospitals NHS Trust, Nottingham, UK), were also sequenced. Where similar isolates were available in our own collection as well as in draft genome form in NCBI, we prioritized the inclusion of isolates from our collection as we had full provenance information (required for the temporal analysis), and we were able to perform phenotypic tests for antimicrobial resistance and additional PCR and sequencing to finish resistance-associated and capsular loci.

DNA was extracted using a protocol for bacteria producing a lot of mucous (capsule) that includes a cetyl trimethylammoninum bromide treatment step (Wilson, 2001). Genomic DNA was retreated with RNase and proteinase K prior to extraction with phenol/chloroform/isoamyl alcohol (25 : 24 : 1) using a Phase Lock Gel (5PRIME) system. Sequencing was performed at the Wellcome Trust Sanger Institute using 96-plex Illumina HiSeq to generate 100 bp paired-end reads as described previously (Harris *et al.*, 2010). Illumina reads are available in the European Nucleotide Archive under project ERP001080; individual accession numbers are given in Table S1. Isolate AB0057 was resequenced as the only available data, a complete genome assembled from 454 reads (Adams *et al.*, 2008), included multiple errors. Sequence yields are given in Table S1.

Isolate A1 was also subjected to PacBio sequencing and manual finishing to obtain a closed reference genome (GenBank accession number CP010781) as described previously (Holt *et al.*, 2015). PacBio sequence data were also used to obtain a closed genome of D36 [GenBank accession numbers CP012952 (chromosome) and CP012953–CP012956 (plasmids)] (Hamidian *et al.*, 2015). Strains AB0057 (Adams *et al.*, 2010), AYE (Vallenet *et al.*, 2008), and AB5075 (Gallagher *et al.*, 2015), for which genome sequences were available but were also examined experimentally for this study, are also listed in Table S1.

### Antimicrobial susceptibility testing

Strains listed in Table S1 were screened for resistance to 20 antibiotics using a disc diffusion assay. Discs (Oxoid) contained ampicillin 25 μg, cefotaxime 30 μg, ceftazidime 30 μg, ticarcillin/clavulanic acid 75/10 μg, imipenem 10 μg, meropenem 10 μg, streptomycin 25 μg, spectinomycin 25 μg, tetracycline 30 μg, trimethoprim 5 μg, sulphonamides 300 μg, amikacin 30 μg, gentamicin 10 μg, kanamycin 30 μg, neomycin 30 μg, netilmicin 30 μg, tobramycin 10 μg, nalidixic acid 30 μg, ciprofloxacin 5 μg and rifampicin 30 μg. Isolates were recorded as resistant according to Clinical and Laboratory Standards Institute guidelines where available (CLSI, 2012) or via comparison to a large pool of susceptible isolates and resistant isolates carrying known resistance determinants.

### SNP detection

Illumina sequence reads were mapped to the A1 reference genome (GenBank accession number CP010781) using the RedDog pipeline (https://github.com/katholt/RedDog). Briefly, reads were mapped using Bowtie2 version 2.2.3 (Langmead & Salzberg, 2012) (using the sensitive-local setting) and SNPs identified from the resulting alignments using SAMtools version 0.1.19 (Li *et al.*, 2009). High-quality SNPs were then extracted using a standard approach as described previously (Holt *et al.*, 2012). For each gene annotated in the A1 reference genome (Holt *et al.*, 2015), RedDog reported its frequency amongst the GC1 genomes, where presence in a genome was defined as ≥ 95 % of the length of the gene being covered by five or more reads. A total of 3312 genes annotated in A1 (91 %) were present in ≥ 90 % of GC1 genomes; these were defined as the core gene set for GC1 and SNPs outside these genes were excluded from phylogenetic analysis. For seven GC1 isolates, the only publicly available sequences were in the form of genome assemblies (see Table S2); hence, in order to include these isolates in the analysis, the assemblies were each shredded into 2 million pairs of reads 100 bp in length (using the wgsim function in SAMtools version 0.1.19) and treated the same way as Illumina reads in the RedDog analysis. The reference genome for GC2 strain 1656-2 (GenBank accession number CP001921) was also included in the same manner in order to provide an outgroup for subsequent phylogenetic analyses.

### Recombination detection and phylogenetic analyses

Pseudo-whole-genome sequences were constructed for each isolate by replacing SNP positions in the A1 reference genome with the consensus allele identified in that isolate from read mapping (minimum Phred score ≥ 20, otherwise replaced by a gap character to indicate an unknown allele or deleted site). These sequences were used to build a pseudo-whole-genome alignment to serve as input to the Gubbins program for recombination detection (Croucher *et al.*, 2014), run with default parameters. Gubbins identifies spatially clustered SNPs that occur on the same branch of the phylogenetic tree, which are likely to have been introduced together via a homologous recombination event (Croucher *et al.*, 2014). This analysis returned an alignment of the non-recombinant SNPs (i.e. substitution mutations) and a maximum-likelihood (ML) phylogeny inferred from these SNPs, and assigns each SNP to either a recombination block or a substitution event and to a branch on the phylogenetic tree. The number of recombination events per 50 bp window was calculated from the blocks output, which lists the coordinates of each recombination block detected and the branch on which it occurred.

**Fig. 1:**
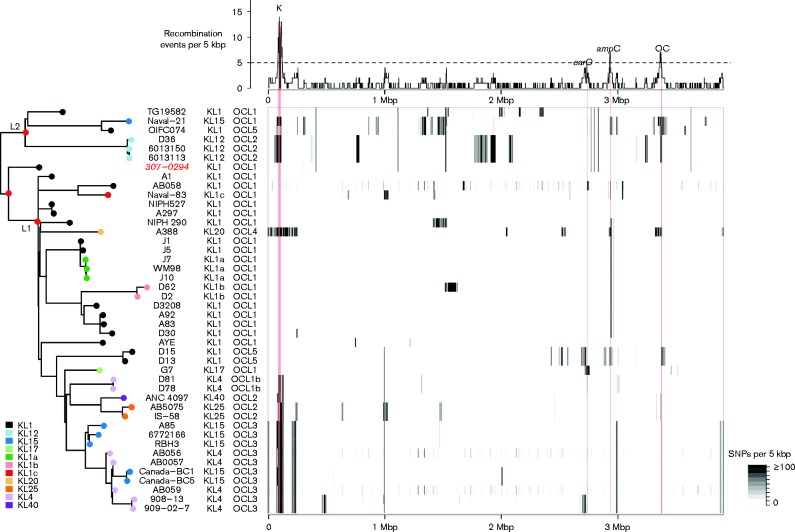
Summary of genome variation in *A. baumannii* GC1. Heatmap (centre): density of SNPs identified in each genome compared with the A1 reference. Plot (top): density of recombination events detected within the GC1 genomes, across the A1 reference genome, inferred from the SNP data using Gubbins. Tree (left): ML phylogeny of genome-wide SNPS, excluding those attributed to recombination events in the Gubbins analysis. L1 and L2, lineages 1 and 2; red nodes have 100 % bootstrap support. Note the isolate highlighted in red has low confidence SNP calls and its position in the tree is uncertain.

The recombination analysis was run with and without the GC2 outgroup, which yielded near-identical results for recombination analysis with Gubbins. We therefore report the results of the Gubbins analysis that included the GC2 outgroup in the alignment, as this allowed the resulting recombination-free phylogenetic tree to be outgroup-rooted. The final ML tree topology and branch lengths (Fig. 1) were inferred from the alignment of substitution mutations output by Gubbins (in which SNP alleles identified as being introduced via recombination were masked by changing them to ‘N’ to indicate an unknown allele) using RAxML with the generalized time reversible (GTR) Gamma model of nucleotide substitution. Ten independent runs of RAxML with 1000 bootstraps each gave near-identical results (including likelihood values, tree topology and branch lengths); thus, we took the first replicate as a single representative result of ML analysis with support values calculated from 1000 bootstraps. The tree topology contained only two bipartitions with < 50 % bootstrap support; these bipartitions were collapsed into polytomies (using TreeCollapserCL 4; http://emmahodcroft.com/TreeCollapseCL.html) to form the final ML tree. The tree and Gubbins output are available in FigShare.

### Selection analysis

Each substitution mutation identified by Gubbins was compared with the annotated A1 genome assembly in order to determine its impact on protein-coding sequences. A total of 1 045 064 codons were annotated in the 3272 core genes; 1504 of these codons harboured substitutions within GC1 (0.14 %), including 14 codons with two independent substitutions (i.e. arising on different branches of the phylogeny) and three codons with four or more independent substitutions. We compared this observed codon frequency spectrum to that expected under a random Poisson distribution (i.e. assuming substitution mutations randomly distributed amongst 1 045 064 codons under the infinite sites model of evolution, with 0.14 % SNPs per codon) (Fig. S1a, b). Under a random distribution of substitutions, we would expect only one codon to harbour more than one substitution mutation; hence, the observed distribution of 17 such codons is much greater than expected under neutral evolution and suggests adaptive selection resulting in convergent evolution. The list of 14 affected genes is given in Table 1. Notably, the codons carrying more than two substitutions include the fluoroquinolone resistance-determining codons in *parC* (six independent non-synonymous substitutions in codon 84) and *gyrA* (four independent non-synonymous substitutions in codon 81). The only other codon in which more than two substitutions were identified was codon 392 of ABA1_02648, which is predicted to encode the receptor for a siderophore (the ferric anguibactin receptor).

**Table 1:** Codons that harbour multiple independent substitution mutations within the GC1 population

Gene	Product	Codon no.	Codon change	Amino acid change	Type*	Note†	Functional note
ABA1_00068	Putative aliphatic sulphonates-binding protein precursor	212	ACA → ACG	T → T	S	( ↔ )	
ABA1_00091 (*gna*)	UDP-*N*-acetyl-galactosamine dehydrogenase	32	GGT → GGC	G → G	S		
			GGA → GGC	G → G	S		
ABA1_00155	BolA family transcriptional regulator	91	TCA → CCA	S → P	NS	( ↔ )	Regulator
ABA1_00226 (*parC*)	ParC DNA topoisomerase IV, A subunit	84	TCG → TTG	S → L	NS	( × 3)	Drug resistance
			TTG → TCG	L → S	NS	( × 2)	
			TTG → TGG	L → W	NS		
ABA1_00716	Hypothetical protein	377	GGA → AGA	G → R	NS	( × 2)	
ABA1_01266	AhpF alkyl hydroperoxide reductase, F subunit	433	GCG → ACG	A → T	NS		
			GCG → GCA	A → A	S		
ABA1_01929 (*acrB*)	RND (resistance-nodulation-division) multidrug efflux transporter	97	CCG → CTG	P → L	NS	( × 2)	Drug resistance, transporter
ABA1_01931 (*adeR*)	Two-component response regulator	26	GAC → AAC	D → N	NS		Drug resistance, regulator
			GAC → GTC	D → V	NS		
ABA1_01953	Major facilitator superfamily transporter, drug : H^+^ antiporter-1	25	TTT → ATT	F → I	NS		Drug resistance, transporter
			TTT → CTT	F → L	NS		
ABA1_02065	PcaH protocatechuate 3,4-dioxygenase, β subunit	144	AAA → ACA	K → T	NS	( × 2)	
ABA1_02141	Proline-specific permease proY	233	CCG → CCA	P → P	S	( × 2)	Transport
ABA1_02431	Serine/threonine protein kinase 1	55	TTT → TAT	F → Y	NS	( × 2)	
ABA1_02631 (*ampC*)	β-Lactamase class C, *ampC*	341	AAC → ACC	N → T	NS	( × 2)	Drug resistance
ABA1_02648	Ferric anguibactin receptor precursor (OM2)	392	GCT → ACT	A → T	NS	( × 3)	Siderophore receptor
			GCT → GAT	A → D	NS		
			GCT → GTT	A → V	NS		
ABA1_02860 (*gyrA*)	DNA gyrase, A subunit	81	TCA → TTA	S → L	NS	( × 4)	Drug resistance
ABA1_03676	Proline-specific permease *proY*	331	AGT → GGT	S → G	NS	( ↔ )	Transport
ABA1_03708	DNA-3-methyladenine glycosylase 1	24	GGC → GGT	G → G	S	( ↔ )	

* NS, non-synonymous; S, synonymous.

† ( ↔ ), both the indicated substitution and its reversion were detected in different parts of the GC1 tree; (x*N*), the indicated substitution mutation was detected multiple times in *N* different branches of the GC1 tree.

Next, we reasoned that the occurrence of substitutions in neighbouring codons could also be a signal of adaptive selection or convergent evolution in a specific region of a gene. We therefore examined the distribution of distances between codons affected by substitutions and compared this with the distribution expected under a random spatial distribution of substitutions in the genome. This revealed an excess of closely spaced substitutions within GC1 (Fig. S1c, d), consistent with adaptive selection in the population. To identify regions of the genome most likely affected by this form of selection, we used a cut-off ratio of observed/expected ≥ 5 to select a distance cut-off for significant spatial clustering. The resulting cut-off was ≤ 5 codons between neighbouring SNPs, i.e. the observed numbers of pairs of SNPs that were separated by one codon, by two codons, etc., up to five codons, were each five or more times that expected under a random distribution of SNPs (Fig. S1c, d); hence, we assume any such pair of SNPs is likely to indicate positive selection in that region of the gene. A total of 66 codons spatially clustered into 39 loci were identified; the 36 affected genes are listed in Table S3. Notably AdeS, AcrB and a third drug efflux gene (ABA1_01953) each harboured two regions with multiple non-synonymous substitutions.

### Temporal analysis

Estimates of the divergence date for GC1 and the substitution rate within GC1 were first made via linear regression of root-to-tip branch lengths (from the final ML tree) on year of isolation using Path-O-Gen version 1.3. Given the strong temporal signal in the data, we proceeded with time-calibrated Bayesian phylogenetic inference using beast version 1.7.5 (Drummond *et al.*, 2012) to analyse the alignment of putative substitution mutations output by Gubbins. Sequences for GenBank accession numbers CP001921 (GC2 outgroup) and TG19582 (uncertain date of isolation) were removed from the alignment, ‘N’ characters introduced by Gubbins to mask recombinant alleles were changed to the gap character ‘-’ so that they were equivalent to unknown base calls, then invariant sites in the alignment were removed. This resulted in an alignment of 1799 SNPs in 44 strains. A best-fit model of nucleotide substitution (GTR) for the alignment was estimated under the Akaike Information Criterion (AIC) using MrModeltest version 2.3 (https://github.com/nylander/MrModeltest2), implemented in paup* version 4.0b10 (http://paup.csit.fsu.edu/). A summary of beast settings is provided in Table S4. All analyses were repeated five times starting from unique random seeds to reduce the chance of accepting runs that had converged on suboptimal likelihoods. Each run was allowed to continue for 50 million iterations, sampling from the posterior every 1000th iteration. Run logs were combined using LogCombiner version 1.7.5 (http://beast.bio.ed.ac.uk) after removing a burn-in of 5 million iterations and resampling every 10 000th iteration (i.e. 22 500 posterior samples drawn from 225 × 10^6^ iterations). Effective sample sizes for all parameters of interest were >1000. Trees were summarized using TreeAnnotator version 1.7.5 (http://beast.bio.ed.ac.uk). The maximum clade credibility tree was annotated with mean heights when posterior support for those branches was >0.5.

We inferred the GC1 time-stamped phylogeny for all samples under both strict (i.e. normally distributed rate variation over the tree) and relaxed (uncorrelated log-normal distribution of rates) clocks. For the strict clock we used a log-normal prior on the clock.rate, with a mean of − 5 and sd of 1.25. For the relaxed clock, we used a log-normal prior on the ucld.mean parameter (which represents the mean substitution rate) with a mean of − 5 and sd of 1.25. As the SNP data were intra-specific, we used a coalescent prior on the tree. Comparisons of clock models were then made using both ‘Bayesian skyline’ (i.e. allowing population size to change over time; complex model) and ‘constant size’ (simple model) tree priors. Using Bayes factor (BF) analysis of the harmonic means of run likelihoods, we found no support for skyline over the constant size (BF < 0.8), so we continued with the simpler, constant-size model. Additionally, as our sampling strategy was opportunistic and not designed for estimating changes in bacterial population size over time, this negated any further exploration using a skyline tree prior. BF analysis showed no support for the relaxed clock over the strict clock (BF < 0.5) and the coefficient of variation for the relaxed clock was ∼0 (9.89 × 10^− 8^), showing no rate variation over the tree. This finding combined with the results of the Path-O-Gen analysis on the ML tree (above) indicated that the strict clock was the most appropriate choice for this dataset. The final beast tree is available in FigShare.

To determine the effect of the outlier sequences on our inferences, we repeated the strict clock analyses after removing the outlier (307-0294; GenBank accession number CP001172) from the alignment. Some invariant sites were introduced into the alignment as a result of removing this sequence, so we reassessed the substitution model with MrModeltest version 2.3 for this reduced alignment. Under the AIC, the GTR remained the most appropriate substitution model.

All analyses were repeated without sequence data (NNNN instead of nucleotide characters) to determine the effect of the priors on the outcome of the analyses. These no-data analyses showed conclusively that the priors alone were not enough to drive our inferences. Additionally, all analyses were repeated without tip-dates, and these recovered near-identical tree topologies (but not branch lengths) to the beast analyses utilizing tip-dates and to the ML analysis. This indicates that the time-calibrated trees are reliable models of ancestor-descendant relationships.

### Genome assembly and analysis of capsule (K) and outer core (OC) loci

For isolates sequenced in this study, Illumina sequence reads were assembled *de novo* using Velvet and Velvet Optimizer, yielding an average of 91 contigs per genome (assembly details and GenBank accession numbers are given in Table S1). For the remaining GC1 genomes, publicly available assemblies were used (GenBank accession numbers are given in Table S2). In each assembly, the exopolysaccharide loci were identified by blastn search for the flanking genes (K: *fkpA*, *lldP*; OC: *ilvE*, *aspS*) as described previously (Kenyon & Hall, 2013; Kenyon *et al.*, 2014b). Each locus was matched against a set of known K loci (Table S5) or OC loci (Kenyon & Hall, 2013; Kenyon *et al.*, 2014b). New K loci were assigned a number (KL#) and the genes within each locus were annotated using the nomenclature system described previously (Kenyon & Hall, 2013).

### Analysis of antimicrobial resistance loci

For the isolates in Table S1, AbaR or Tn*6019* : : Tn*6018* regions in *comM* were published or were mapped as described previously (Post *et al.*, 2010). Associated contigs were retrieved, and the sequence assembled and finished using capillary sequencing of linking PCR products (see references for AbaR types in Tables S1 and S2). Plasmids carrying antibiotic resistance genes were assembled using PCR and sequencing the products as described previously (Hamidian *et al.*, 2014a, b). The presence of IS*Aba1* or IS*Aba125* upstream of the *ampC* gene and of the presence of Tn*6168* was established using PCR and capillary sequencing as described previously (Hamidian *et al.*, 2013; Hamidian & Hall, 2014b). All antimicrobial resistance genes in all genomes were identified by a combination of automated screens and manual curation and assembly of known resistance regions. Automated analyses included screening for acquired resistance genes in the reads using srst2 (Inouye *et al.*, 2014) and in the assemblies using blastn, analysis of IS*Aba1* and IS*Aba125* insertions directly from reads using ISMapper (Hawkey *et al.*, 2015), and mapping of reads to known resistance elements, plasmids and transposons using the RedDog pipeline as described earlier under SNP detection (https://github.com/katholt/RedDog).

## Results

### Population structure within GC1

We analysed the genomes of a total of 45 *A. baumannii* GC1 isolates, spanning the 30-year period from 1982 to 2011. Twenty-three genomes were sequenced for this study (isolates selected to maximize diversity with respect to time of isolation and antimicrobial resistance profile) and the remainder were publicly available (Tables S1 and S2). The genome of the oldest available GC1 isolate A1 (1982, UK; Hamouda *et al.*, 2010) was finished using a combination of Illumina, PacBio and capillary sequencing (Holt *et al.*, 2015) to serve as the reference for comparative genomic analyses. A total of 25 752 high-quality SNPs were identified amongst the GC1 isolates. Analysis with Gubbins (Croucher *et al.*, 2014) predicted 24 354 SNPs were associated with 134 homologous recombination events affecting 31/44 branches of the phylogeny (see Methods) (Fig. 1).

The ratio of SNPs introduced by recombination compared with mutation (*r*/*m*) was 22 and the ratio of recombination events compared with substitution mutations (ρ/θ) was 0.1. Four hotspots of recombination, affected by at least five recombination events each, were identified within GC1 (Fig. 1). Three were associated with surface structures: biosynthesis of exopolysaccharides via the K locus and OC locus, and the gene encoding the outer membrane protein CarO. The fourth introduces resistance to third-generation cephalosporins via the insertion sequence-enhanced expression of the intrinsic AmpC β-lactamase. Thirty-six genes harboured a surplus of independent substitutions in the same or neighbouring codons, consistent with convergent evolution (see Methods) (Fig. S1). These genes, listed in Tables 1 and S3, include several with a role in antimicrobial resistance (*gyrA*, *parC*, penicillin-binding protein, and multidrug efflux pumps encoded in the *acr* and *ade* clusters), as well as genes involved in capsule expression (*wzc*), membrane transport, stress response, and synthesis of fimbriae and siderophore receptors.

**Fig. 2:**
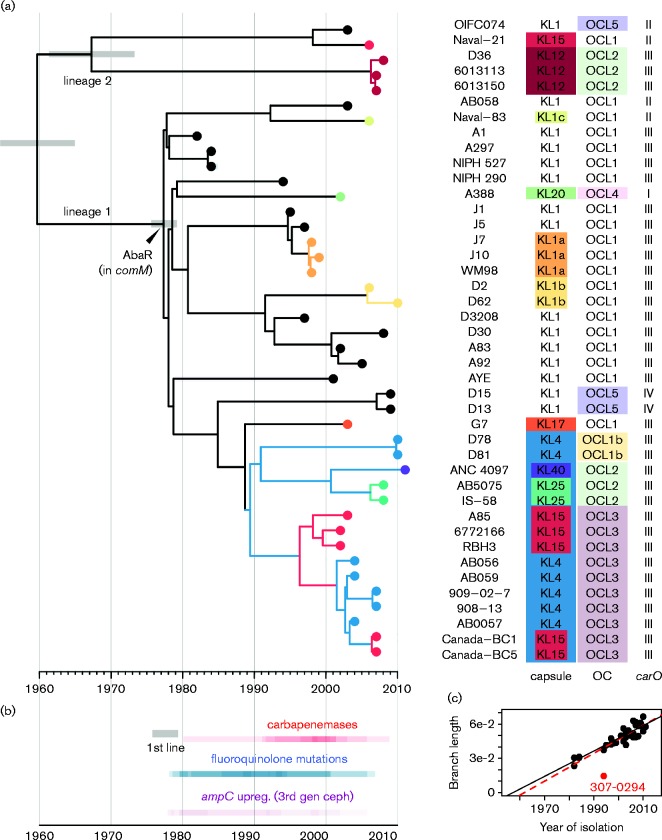
Temporal and phylogenetic analysis of 43 *A. baumannii* GC1. (a) Dated whole-genome phylogeny constructed using beast. *x*-axis, calendar years; grey bars, 95 % highest posterior density for divergence dates of selected nodes; tip colours, KL types; branch colours, clades sharing a recombination event resulting an exchange of the KL. Tips are labelled with isolate names and the KL and OC types, coloured to highlight those that differ from the inferred ancestral types KL1 and OCL1. (b) Heated timeline indicating time intervals in which antimicrobial resistance-associated mutations arose, inferred from the tree by overlaying *x* (time) coordinates of branches on which the mutational events occurred. Upreg. (3rd gen ceph), upregulation of *ampC* gene expression resulting in resistance to third-generation cephalosporins. (c) Scatter plot showing linear relationship between year of isolation and ML branch lengths (tree shown in Fig. 1). Red point, isolate for which genome data may be unreliable (excluded from beast analysis); black line, regression line excluding this isolate; red line, regression line including all isolates.

We inferred a ML phylogenetic tree from the alignment of ∼2000 substitution mutations (SNPs not associated with recombination events) (Fig. 1), rooted using a GC2 outgroup. There was a strong linear relationship between year of isolation and ML root-to-tip branch lengths (*R* ^2^ = 0.73), indicating a strong molecular clock with the accumulation of ∼5 SNPs genome^− 1^ year^− 1^ (equivalent to 1.5 × 10^− 6^ substitutions site^− 1^ year^− 1^) and a most recent common ancestor (MRCA) for GC1 around 1960 (Fig. 2c). One of the complete genomes, 307-0294, was an outlier in the year versus branch length plot (Fig. 2c). We consider this likely due to sequencing errors (only an assembly of 454 reads was available) rather than true rate variation; hence, this genome and that of TG19582 (isolation date unknown) were excluded from temporal analysis, and its position in the ML tree should be considered of low confidence.

The 43 high-quality genome sequences with known isolation dates were subjected to Bayesian analysis using beast. The resulting tree topology and divergence date estimates were consistent with those from ML analysis [GC1 MRCA around 1960 (95 % highest posterior density (HPD) 1954–1965] (Figs 2a and S2, Table S4). The trees showed GC1 has diverged into two major lineages, 1 and 2, each with 100 % clade support in both ML and Bayesian analyses (Figs 1, 2 and S2). Lineage 1 included 37 of the sequenced isolates, which shared an MRCA around 1977 (95 % HPD 1975–1979), separated from the GC1 MRCA by 45 SNPs (Fig. 2). The oldest GC1 strains in our collection, isolated in 1982 and 1984, were separated from the MRCA of GC1 lineage 1 by 21–24 non-recombinant SNPs. In total, 1355 non-recombinant SNPs were identified within GC1 lineage 1, many of which belonged to long branches consistent with divergence into multiple successful subclones (Fig. 2). Lineage 2 included five strains isolated from soldiers in the US, UK and Australia between 2003 and 2008, and the isolate TG19582, about which little information is available. Lineage 2 had a MRCA around 1967 (95 % HPD 1961–1973), separated from the GC1 MRCA by 49 SNPs, and was divided into multiple sublineages (Figs 1, 2 and S2). A possible third GC1 lineage was represented by the single genome 307-0294. Though excluded from the main analysis (as outlined earlier), analyses including this strain indicated it may have diverged from lineage 1 shortly after its divergence from lineage 2 (Fig. 1).

### Antibiotic resistance in lineage 1

**Fig. 3:**
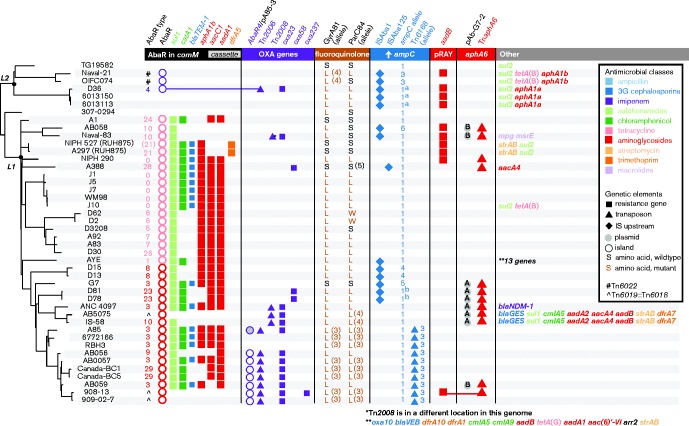
ML tree and antimicrobial resistance determinants for 44 *A. baumannii* GC1. L1 and L2, lineage 1 and 2. Key resistance determinants are indicated with symbols, according to inset legend.

All lineage 1 genomes carried a resistance island (AbaR) with a common Tn*6019* backbone within the *comM* gene or Tn*6019* : : Tn*6018*, a derivative from which all antibiotic resistance genes have been lost (Figs 2, 3 and S3). These data support a single introduction of the multiple antibiotic resistance island into this lineage in the late 1970s, consistent with anecdotal evidence of the emergence of multidrug-resistant *A. baumannii* at this time (resistant to ampicillin, sulphonamides, tetracycline, gentamicin and kanamycin, and nalidixic acid) (Bergogne-Bérézin & Towner, 1996) and the detection of an AbaR-carrying isolate in 1977 (Devaud *et al.*, 1982; Krizova & Nemec, 2010). The phylogeny supports our previous report that the initial insertion was of an AbaR0-type backbone (Hamidian *et al.*, 2014c), and that the deletion in the *intI1* gene that defines AbaR3 (location shown by the vertical arrow in Fig. S3a) arose once and is a phylogenetically informative marker (Fig. 3). Subsequent microevolution of AbaR0 and AbaR3 included insertions and deletions of antimicrobial resistance genes, as well as loss of parts of the AbaR backbone, mostly due to IS*26*-mediated deletions (Post & Hall, 2009; Post *et al.*, 2010, 2012; Krizova *et al.*, 2011; Nigro *et al.*, 2011b). This resulted in many variants (Fig. 3, Tables S1 and S2).

Plasmids have also contributed to aminoglycoside resistance and both pRAY*, carrying the *aadB* gentamicin/tobramycin resistance gene (Hamidian *et al.*, 2012), and conjugative plasmids of the *rep*Aci6 group, carrying the *aphA6* amikacin resistance gene in Tn*aphA6* (Hamidian *et al.*, 2014a), were found in lineage 1 (Fig. 3).

Our data demonstrated that resistance to antibiotics introduced in the 1980s, including fluoroquinolones, third-generation cephalosporins and carbapenems, emerged multiple times within GC1 via numerous distinct substitution, recombination, transposition and plasmid acquisition events (Fig. 3). The dated phylogeny indicated that resistance-conferring mutational events began to arise within GC1 lineage 1 in the 1980s, shortly after the acquisition of the AbaR0 resistance island. The frequency of acquisition of carbapenem resistance genes intensified in the 1990s (Fig. 2b).

Resistance to newer antibiotics arose in several ways. Fluoroquinolone resistance arose via substitution mutations in *gyrA* and *parC* or by import of an allele that contains the relevant mutations. In lineage 1, this occurred once for *gyrA* and twice for *parC* (Fig. 3). A third *parC* exchange in isolate A388 is associated with a susceptible allele. Resistance to third-generation cephalosporins arose in various ways, all involving an upstream insertion sequence that increased expression of the intrinsic *ampC* allele. These were incorporation of IS*Aba125* or IS*Aba1* upstream of the original *ampC* allele (Hamidian & Hall, 2013; Hamidian *et al.*, 2013), introduction of a novel *ampC* allele together with IS*Aba1* (Hamidian & Hall, 2014a), and introduction of transposon Tn*6168*, carrying an additional *ampC* gene, flanked IS*Aba1* (Hamidian & Hall, 2014b). Resistance to carbapenems was most often caused by the introduction of the *oxa23* gene in Tn*2008* (in two separate locations; Nigro & Hall, 2015, 2016) or Tn*2006* in Tn*6022* (AbaR4) (Fig. S3). AbaR4 was found both in the chromosome (Adams *et al.*, 2008) and on a conjugative *rep*Aci6 plasmid (Hamidian *et al.*, 2014b). However, *oxa58*, *oxa237* and *bla*_NDM-1_ genes were also detected (Fig. 3).

### Antibiotic resistance in lineage 2

In contrast to lineage 1, GC1 lineage 2 genomes either have no transposon in *comM* or have acquired a different transposon, Tn*6022*, which does not carry any antibiotic resistance genes (Fig. S3), at this site. In one case, a Tn*2006* carrying the *oxa23* carbapenemase resistance gene had inserted into Tn*6022* to form AbaR4 (Fig. S3) (Hamidian & Hall, 2011). Hence, this isolate was the only lineage 2 isolate exhibiting carbapenem resistance. However, all of these isolates carried the *sul2* sulphonamide resistance gene (Fig. 3). A plasmid related to pD36-4 (GenBank accession number CP012956) (Hamidian *et al.*, 2015) that carried *sul2* and also carried an *aphA1a* kanamycin and neomycin resistance gene in Tn*4352* was also present in two closely related isolates. The location of *sul2* in the remaining lineage 2 isolates was not known. Plasmids have also contributed to gentamicin and tobramycin resistance, particularly pRAY* carrying *aadB* (Hamidian *et al.*, 2012), which was found in both lineages. Naval-21 and OIFC074 also carried a *tetA*(B) tetracycline resistance determinant and an *aphA1b* gene, but their context remains to be determined.

In lineage 2, resistance to fluoroquinolones also arose via mutations in *gyrA* and *parC* or import of a novel allele. Resistance to the third-generation cephalosporins arose when *ampC* was activated by IS*Aba1* and via import of an insertion sequence-activated allele from elsewhere.

### Replacement of the K and OC gene clusters

**Fig. 4:**
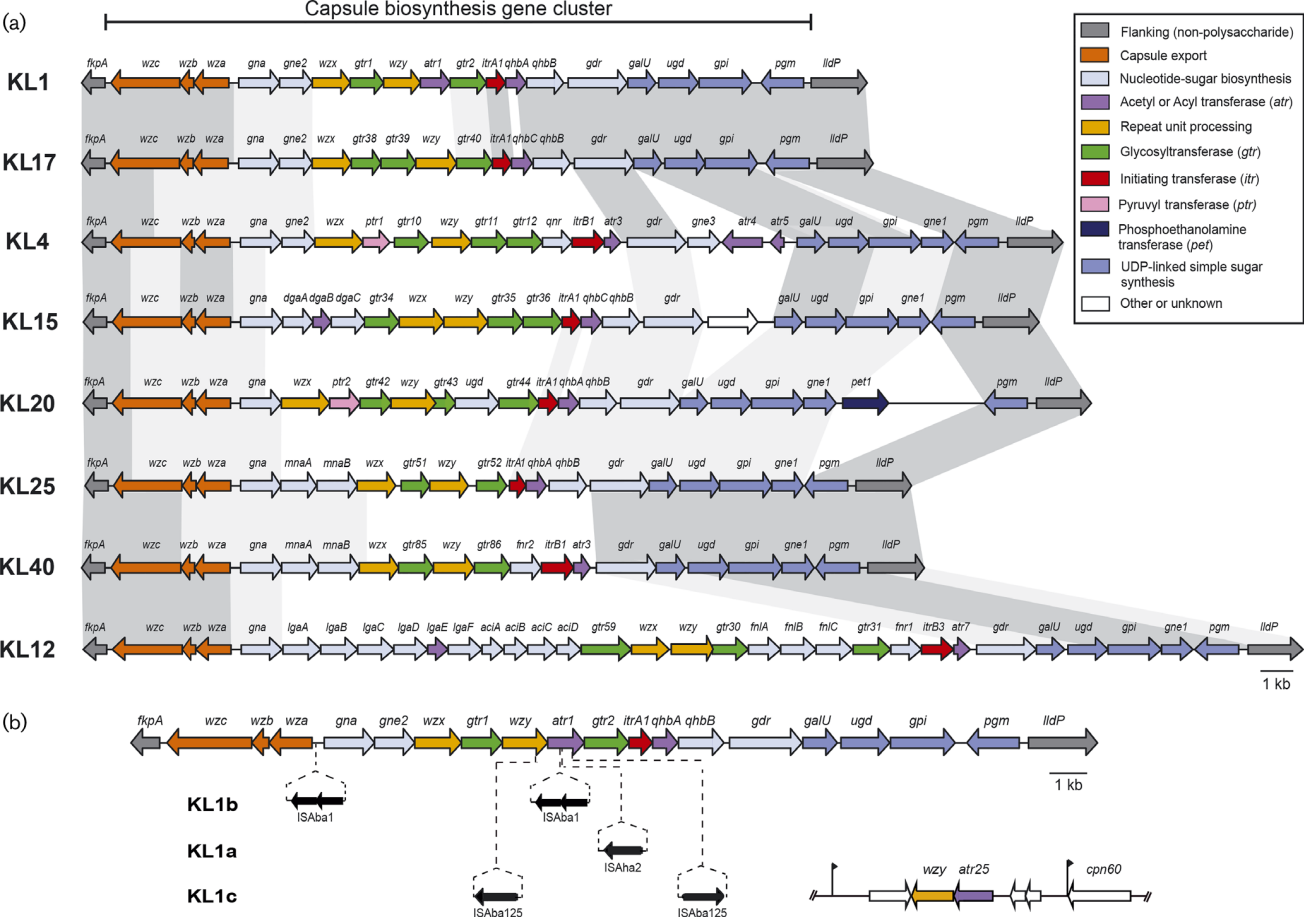
Capsule biosynthesis gene clusters (KL) in GC1 genomes. Capsule gene clusters are drawn to scale from GenBank accession numbers listed in Table S1 (and GenBank accession number KP100029 for KL40), scale bar is shown. The horizontal bar above indicates the K locus region and the KL names are shown on the left. Genes are shown as arrows that are coloured according to the biological role of the encoded product, according to the colour scheme shown on the right. (a) Alignment of GC1 capsule gene clusters. Dark grey shading between gene clusters indicates DNA sequence that is >95 % identical, light grey shading indicates 80–95 % DNA identity. (b) Insertion variants of KL1. Positions of insertion sequence elements in each KL1 variant are shown with the identity and orientation of the insertion sequence below. For KL1c in Naval-83, the additional segment containing *wzy*, *atr25* and three unknown ORFs and its position in the genome is shown on the right. Black flags indicate the position of a 9 bp duplication.

A total of eight distinct gene clusters directing the synthesis of capsule (K) (Fig. 4, five of which had been noted previously; Kenyon & Hall, 2013; Hamidian *et al.*, 2014b; Kenyon *et al.*, 2014a, 2015) and five gene clusters for synthesis of the outer core (OC) of lipooligosaccharide (Kenyon & Hall, 2013; Kenyon *et al.*, 2014b) were identified amongst the GC1 genomes (Fig. 2). The KL1 locus was present throughout the GC1 phylogenetic tree, in both lineages, and was in all strains isolated up to 2001 (Tables S1 and S2). A similar pattern was observed for OCL1 (Fig. 2). Thus, we infer the MRCA of GC1 most likely carried KL1 and OCL1, and produced the K1 capsule type and OC1 outer core type. This was supported by ML ancestral trait reconstruction analysis, treating KL and OCL types each as discrete traits (Fig. S4). The earliest KL variant in our collection was KL1a, derived from KL1 via the insertion of IS*Aha2* in an acetyltransferase-encoding gene (Fig. 4b), and was found in Australian lineage 1 isolates from 1998 that also carried OCL1 (Fig. 2). Two other unrelated insertion sequence-mediated KL1 variants, KL1b and KL1c, were also observed (Fig. 4b), all in isolates carrying OCL1 (Fig. 2). In the Naval-83 isolate carrying KL1c, an additional *wzy* gene inserted elsewhere in the chromosome replaced the interrupted *wzy* gene (Fig. 4b).

**Fig. 5:**
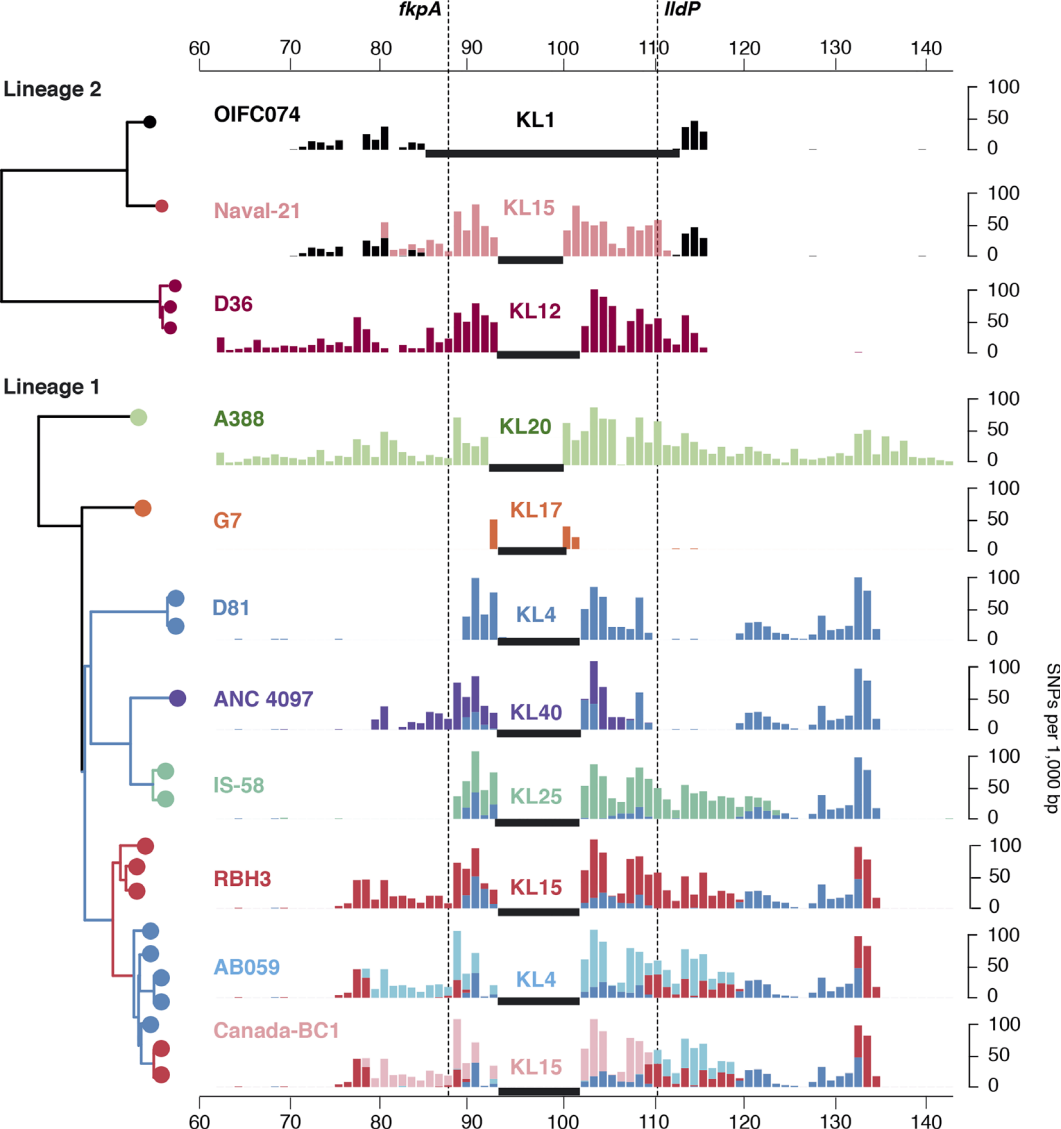
Recombination events affecting KL in GC1. Left: tree showing relationships between isolates carrying KL types other than the ancestral type (KL1), extracted from the whole-of-GC1 beast tree (Fig. 3). Leaf nodes are coloured by KL type, branch colours indicate shared ancestry of the K locus (inferred from shared SNPs shown in the bar plot). Bar plots: density of SNPs compared with the A1 reference genome; black horizontal bars, regions that are non-homologous with A1 and thus no SNPs can be called; shared SNP alleles are indicated by use of the same colours across plots (i.e. each colour represents a single recombination event). *x*-axis, coordinates (Mbp) in the A1 reference genome; dashed lines indicate KL boundaries.

**Fig. 6:**
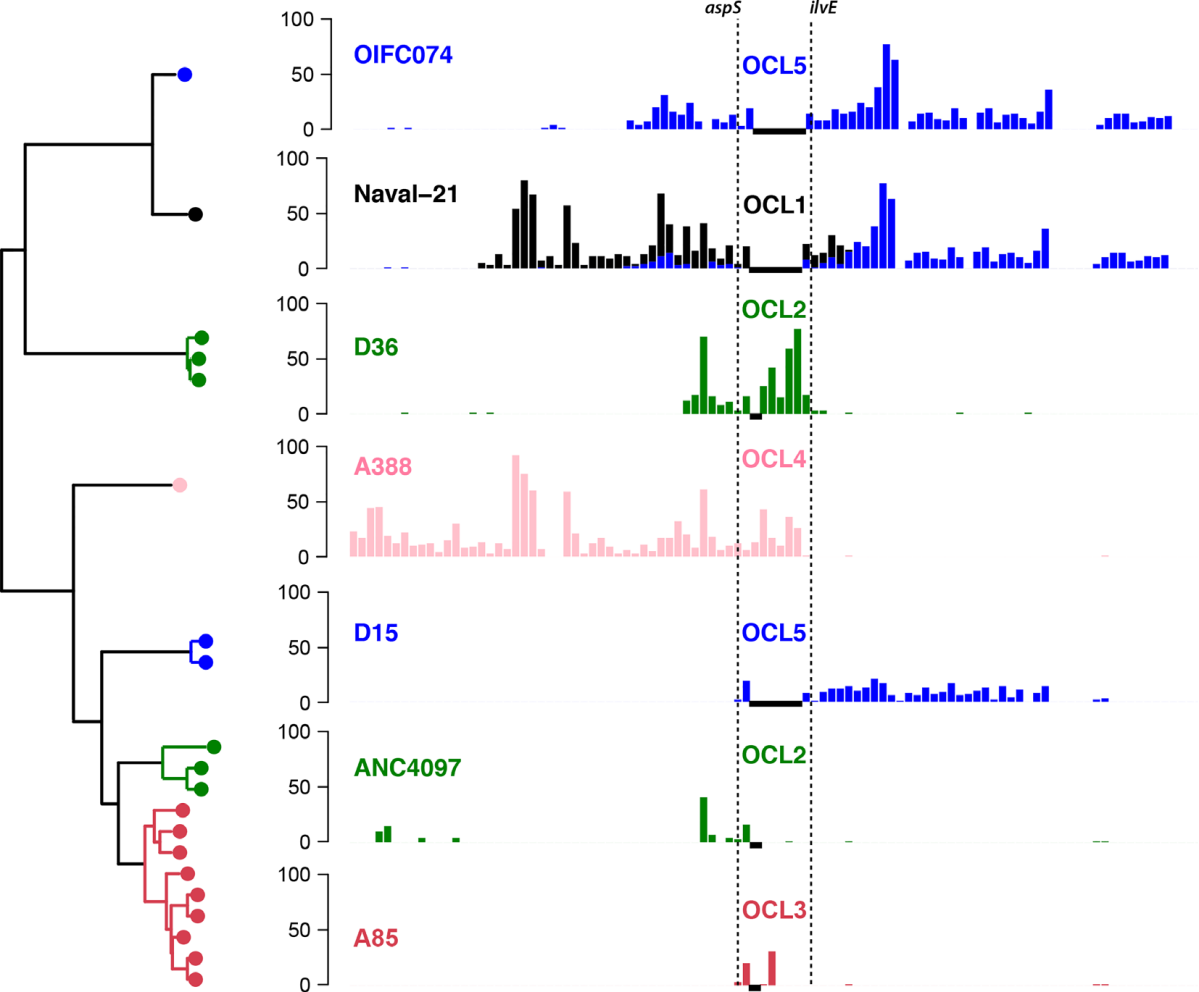
Recombination events affecting the OCL in GC1. Left: tree showing relationships between isolates carrying OCL types other than the ancestral type (OCL1), extracted from the whole-of-GC1 beast tree (Fig. 3). Leaf nodes are coloured by OCL type, branch colours indicate shared ancestry of the OC locus (inferred from shared SNPs shown in the bar plot). Bar plots: density of SNPs compared with the A1 reference genome; black horizontal bars, regions that are non-homologous with A1 and thus no SNPs can be called; shared SNP alleles are indicated by use of the same colours across plots (i.e. each colour represents a single recombination event). *x*-axis, coordinates (Mbp) in the A1 reference genome; dashed lines indicate OCL boundaries.

Non-KL1 (and non-OCL1) loci are clustered within the GC1 phylogenetic tree, resulting in phylogenetically distinct subclones of GC1 with different surface polysaccharides. Analysis of shared SNPs identified a total of 10 recombination events spanning the K locus, resulting in capsular exchange (Figs 2 and 5). In our collection, the earliest examples of non-KL1 capsules were isolated in 2002 (isolate A388, 263 kbp exchange introducing KL20) and 2003 (isolate G7, 55 kbp exchange introducing KL17) (Fig. 5). However, we detected a 45 kbp import of KL4 that was shared by a lineage 1 subclade whose MRCA was estimated around 1987 (blue branches in Figs 2 and 5), suggesting that capsular exchanges began occurring much earlier. Within this KL4 subclade of lineage 1, we detected a further five recombination events resulting in capsular exchange (one to KL40; one to KL25; one to KL15, then back to KL4, then back to KL15; Fig. 5) and two resulting in OCL exchange (one to OCL2 and one to OCL3; Fig. 6). In total, we observed five distinct OC loci in GC1, OCL1–5 (structures reported elsewhere; Kenyon *et al.*, 2014b), which we traced to seven recombination events (Figs S4 and 6).

## Discussion

Our analysis indicates that the MRCA of *A. baumannii* the GC1 clonal complex arose around 1960 and later diverged into two phylogenetically distinct lineages. In the mid-1970s, the main lineage acquired resistance to older antibiotics via a single event creating the AbaR0 resistance island in the chromosome and giving rise to the multiply antibiotic-resistant lineage 1. Later, a sublineage carrying AbaR3, identifiable by a small deletion in *intI1*, emerged (Hamidian *et al.*, 2014c). Both sublineages of lineage 1 have since given rise to multiple successful subclones, by losing some of the resistance genes in AbaR and accumulating resistance to newer antibiotics through various genetic mechanisms, including the acquisition of plasmids and transposons or acquisition of mutations in chromosomal genes. Ultimately, this has given rise to isolates that are extensively antibiotic-resistant, potentially belonging to many different sublineages. Lineage 2 currently includes only a few military isolates, indicating they are likely to have originated in Afghanistan or Iraq, and has acquired resistance via different routes. In the future, detailed tracking of the spread of GC1 within hospitals, cities, countries and globally will need to entail tracking of specific lineages, and the subclones and sublineages of them. PCR assays to distinguish different gene clusters at the K locus similar to those reported for the OC locus (Kenyon *et al.*, 2014a) should assist this. More detailed information on the plasmids carrying antibiotic resistance genes will also be valuable.

Capsule replacement has been extensively studied in *Streptococcus pneumoniae*, where it is associated with immune evasion and vaccine escape. However, the capsular exchange detected here within the recently emerged *A. baumannii* GC1 clone adds to the occasional exchanges reported previously in GC1 (Adams *et al.*, 2008; Kenyon & Hall, 2013; Hamidian *et al.*, 2014b; Kenyon *et al.*, 2015), bringing the total distinct KL gene clusters to eight, similar to that reported previously in the GC2 clone (Snitkin *et al.*, 2011; Hu *et al.*, 2013; Kenyon & Hall, 2013; Wright *et al.*, 2014). The number of capsule replacement events in these *A. baumannii* groups is significant amongst Gram-negative epidemic clones. O-antigen or capsule replacement has recently been reported in lineages of *E. coli*, including 10 capsular types within *E. coli* ST131 (Iguchi *et al.*, 2015; Alqasim *et al.*, 2014; Riley, 2014), and a capsule replacement event has been noted within *K. pneumoniae* epidemic clone ST258 (DeLeo *et al.*, 2014; Wyres *et al.*, 2015). Notably, our analysis indicates that the process of replacement of the gene cluster at the KL in GC1 did not begin until after the emergence of the two successful lineages 1 and 2, following the independent acquisition of multiple antimicrobial resistance determinants into the chromosome of both lineages. It was recently reported that the evolution of multidrug-resistant O12 *Pseudomonas aeruginosa* involved a change from the O4 serotype via recombination (Thrane *et al.*, 2015), which also introduced novel antimicrobial resistance genes; we speculate that further exchanges are likely to occur as this clone diversifies.

The extensive KL and OCL replacement detected in our collection is suggestive of selection for phenotypic variation in exopolysaccharides expressed by GC1; however, the evolutionary drivers of capsular exchange in *A. baumannii* are not yet clear. It is tempting to speculate that host immunity is a key driver of capsular exchange and this could help explain the apparent increase in capsular changes over time in the GC1 population as it adapts to the new niche of a human-associated hospital pathogen following the acquisition of multiple antibiotic resistance. However, the variation we observed may be associated with factors unrelated to host immunity, including diversifying selection from predatory bacteriophage and amoeba that recognize specific capsule types (Adiba *et al.*, 2010), or with subtle differences in antimicrobial susceptibility that have also recently been linked to capsular variation (Geisinger & Isberg, 2015).

Whatever the drivers, it is likely that similar selective pressures are at play in the more recently emerged multidrug-resistant epidemic clones *E. coli* ST131 and *K. pneumoniae* CC258, in which relatively limited capsular exchange has so far been documented. This has important implications for the prospect of capsule-targeted vaccines and phage therapy, which have been proposed as a control measure for highly drug-resistant hospital outbreak-associated clones of *A. baumannii* and *K. pneumoniae* (Ahmad *et al.*, 2012; García-Quintanilla *et al.*, 2013; Russo *et al.*, 2013).

Our analysis traces in fine detail the myriad microevolutionary events that have accompanied the emergence of multidrug-resistant *A. baumannii* GC1 over the last 65 years as it accumulated resistance to first-line antimicrobials, then fluoroquinolones, third-generation cephalosporins and carbapenems. The revelation of extensive capsule replacement during this period, resulting in a diversity of GC1 subclones with distinct surface polysaccharides and antimicrobial resistance phenotypes, has significant implications not only for epidemiological tracking and hospital infection control, but also for the development of novel vaccines, therapeutics and diagnostics targeting this extremely drug-resistant and globally distributed clone. Further, these patterns provide a glimpse into the future evolutionary trajectory of the much more recently emerged epidemic clones of *Enterobacteriaceae*, which show similar signs of capsular diversification and accumulation of complex resistance determinants.

## Supplementary Data

1840 Supplementary Data
