# Corrigendum: Five decades of genome evolution in the globally distributed, extensively antibiotic-resistant *Acinetobacter baumannii* global clone 1

**DOI:** 10.1099/mgen.0.000280

**Published:** 2019-07-31

**Authors:** Kathryn E. Holt, Johanna J. Kenyon, Mohammad Hamidian, Mark B. Schultz, Derek J. Pickard, Gordon Dougan, Ruth M. Hall

**Affiliations:** ^1^​ Department of Biochemistry & Molecular Biology, The University of Melbourne, Royal Parade, Parkville, Victoria, Australia; ^2^​ School of Biomedical Science, Queensland University of Technology, Brisbane, Queensland, Australia; ^3^​ School of Molecular Bioscience, The University of Sydney, Sydney, New South Wales, Australia; ^4^​ Centre for Systems Genomics, The University of Melbourne, Parkville, Victoria, Australia; ^5^​ Wellcome Sanger Trust Institute, Hinxton, Cambridge, UK

## Full Text

There was a change in the author names in the published article. The new list should read:

Kathryn E. Holt^1^, Johanna J. Kenyon^2^, Mohammad Hamidian^3^ , Mark B. Schultz^4^, Derek J. Pickard^5^, Gordon Dougan^5^, Ruth M. Hall^3^

1, Department of Biochemistry & Molecular Biology, The University of Melbourne, Royal Parade, Parkville, Victoria, Australia

2, School of Biomedical Science, Queensland University of Technology, Brisbane, Queensland, Australia

3, School of Molecular Bioscience, The University of Sydney, Sydney, New South Wales, Australia

4, Centre for Systems Genomics, The University of Melbourne, Parkville, Victoria, Australia

5, Wellcome Sanger Trust Institute, Hinxton, Cambridge, UK

The author apologizes for any inconvenience caused.

