# Supplementary material for: Five decades of genome evolution in the globally distributed, extensively antibiotic-resistant Acinetobacter baumannii global clone 1
Source: Microb Genom. 2016 Feb 23;2(2):e000052. doi: 10.1099/mgen.0.000052 (PMC5320584; doi:10.1099/mgen.0.000052)
Supplement: Supplementary file 1 — Supplementary Data [file mgen-02-52-s001.pdf]

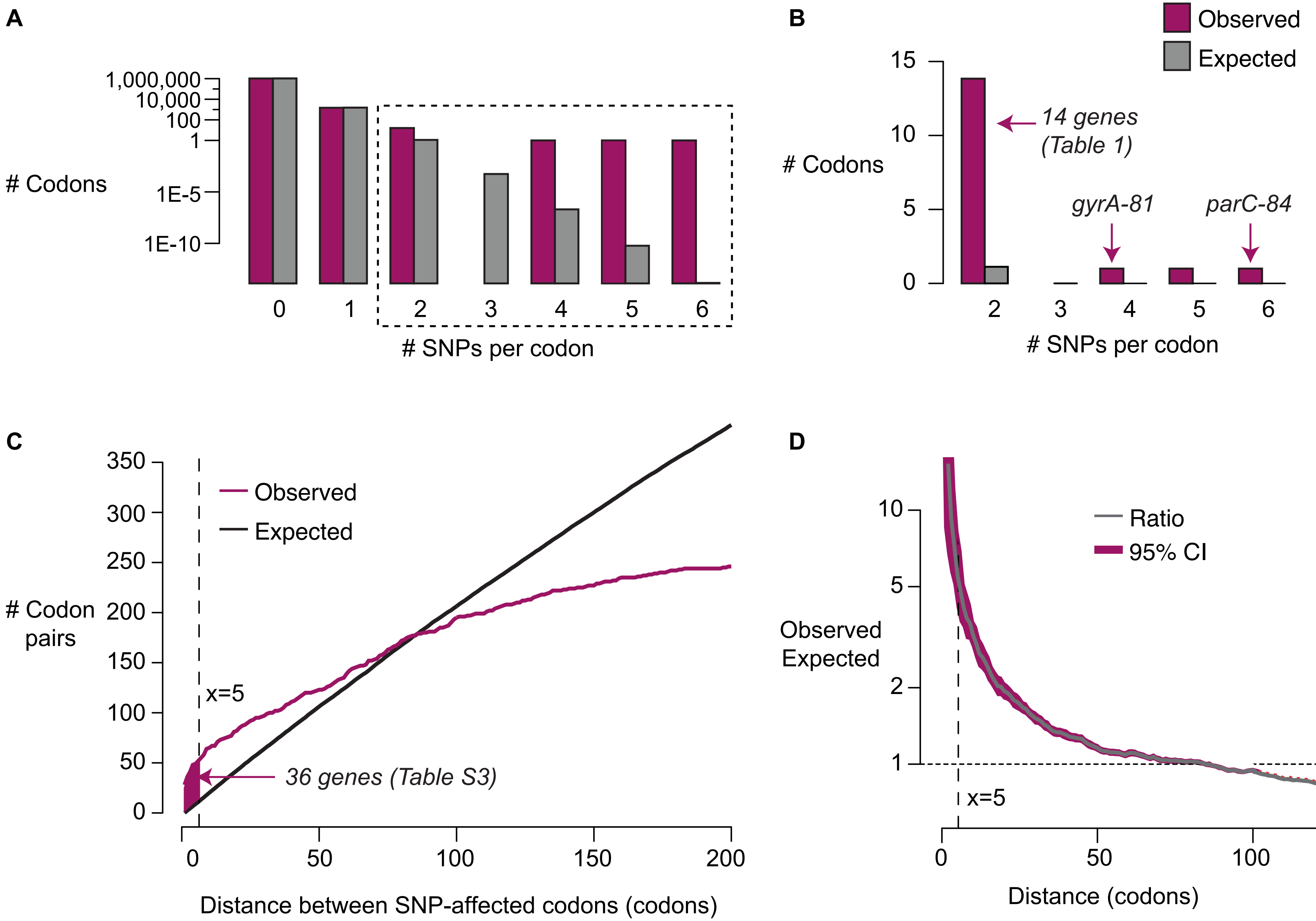

Figure S1. Selection analysis of *A. baumannii* GC1 based on SNP variation.
(A-B) Codon mutation frequency spectrum, shown in log scale **(A)** and ordinal scale for zoom-in on codons with ≥2 SNPs **(B)**. Expected values were calculated assuming a random Poisson distribution of SNPs (see **Methods**). Affected genes are detailed in **Table 1**.
(C-D) Frequency distribution of distances between SNP-affected codons, expected values calculated assuming a random spatial distribution of SNPs in the chromosome. Shaded region in **(C)** indicates SNP distances that are 5-fold enriched compared to the random distribution, which happens to correspond to a distance of ≤5 codons (as shown in **D**). That is, there are 5 times as many SNP pairs separated by 5 or fewer codons than would be expected under a random spatial distribution of SNPs. This is taken to indicate significant clustering of SNPs over small distances; the affected genes are listed in **Table S3**.

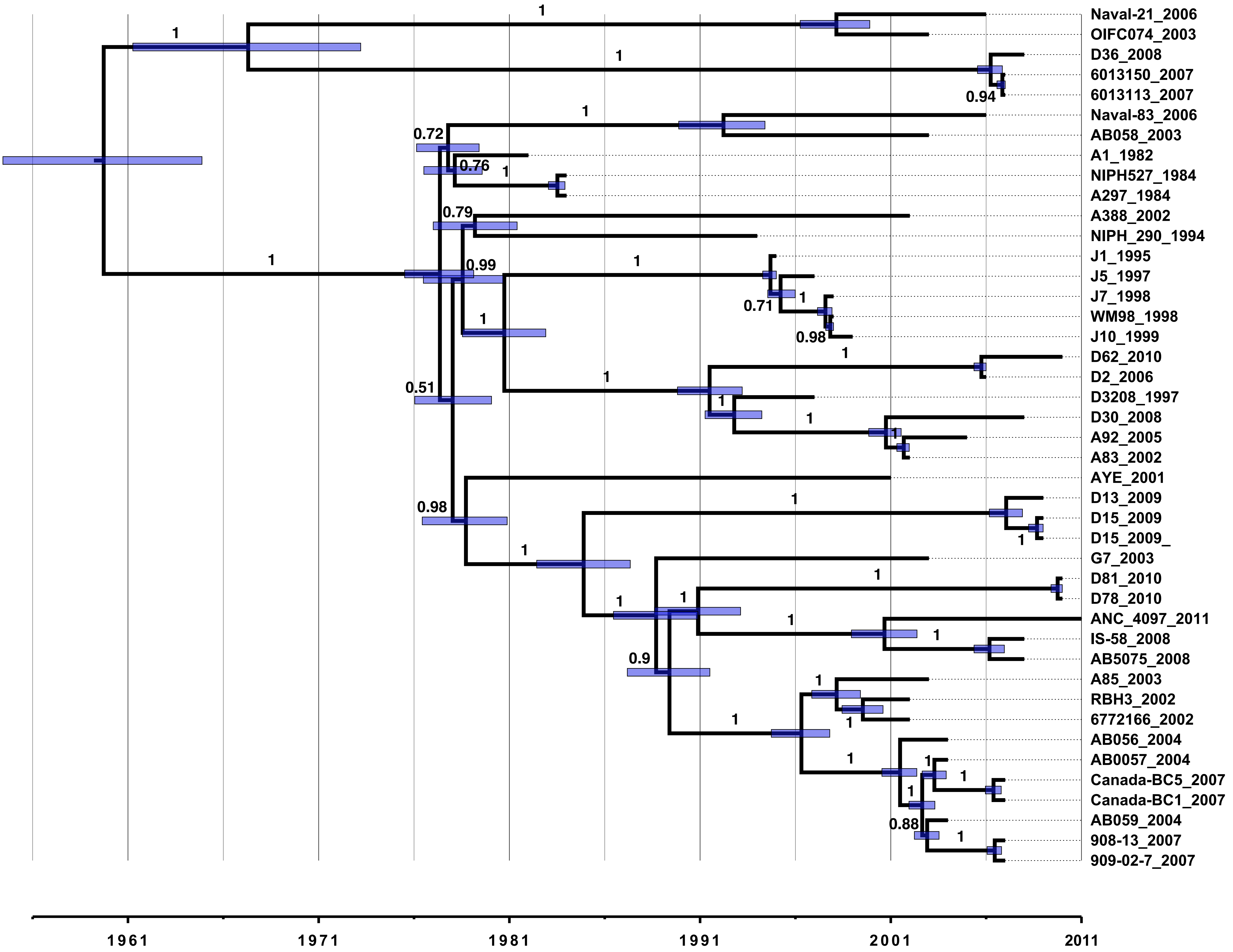

Figure S2. Phylogeny inferred using BEAST.

Blue bars indicate 95% HPDs for node dates. Branches are labeled with clade support.

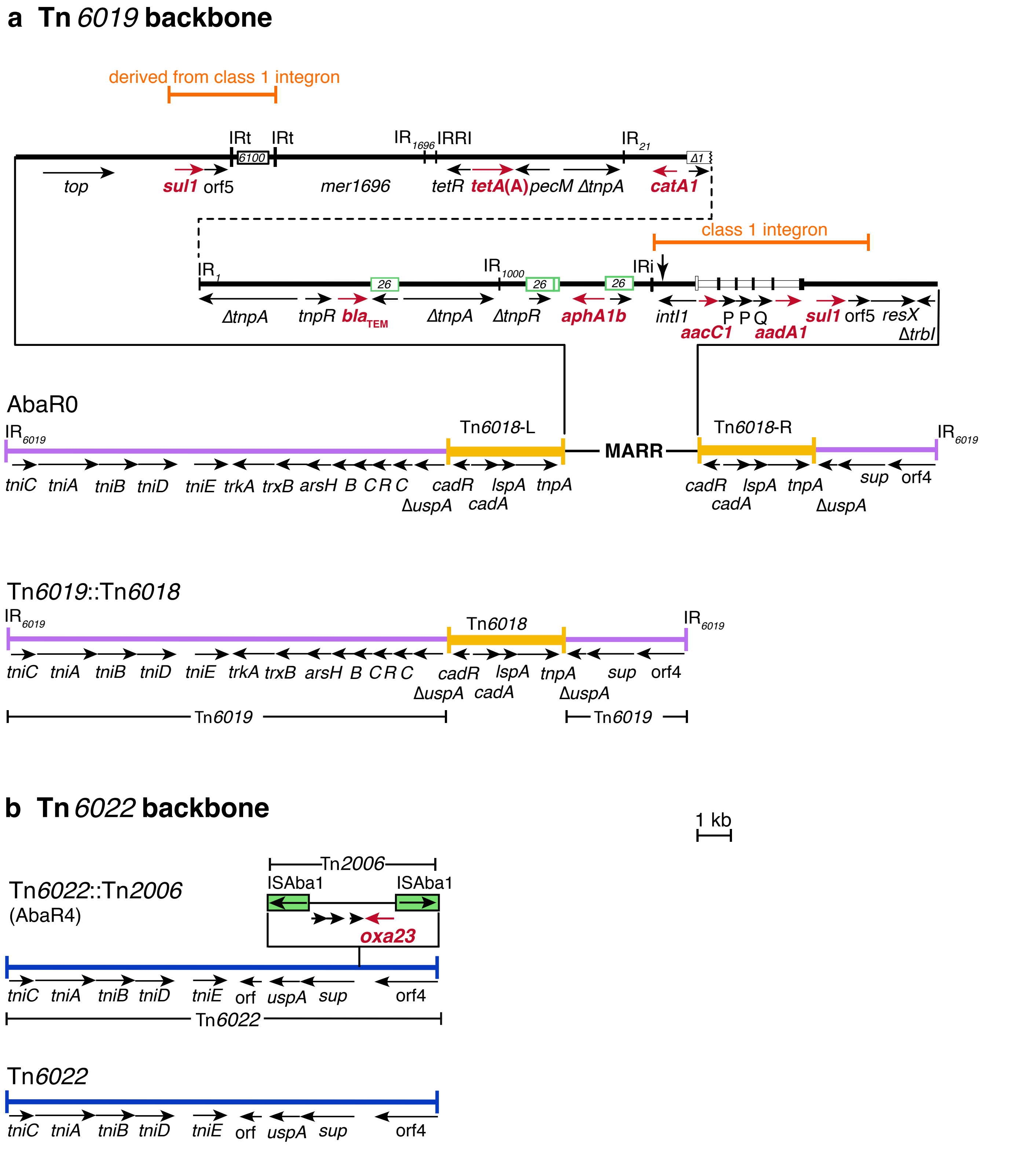

Figure S3. Structures of transposons found in *comM*.

A. Transposons with a Tn*6019* backbone. Segments derived from Tn*6019* are mauve and Tn*6018* are ochre; inverted repeats (IR) shown as vertical lines. The structure of the Multiple Antibiotic Resistance Region (MARR) of AbaR0 is shown above. Labeled arrows below show the orientation and extent of genes and open reading frames, and antibiotic resistance genes are red. Genes conferring resistanc to mercuric ions are represented by *mer1696*. Open boxes with a number inside represent IS (insertion sequences) and open boxes with vertical bar at their ends represent gene cassettes. The extents of the two class 1 integron-derived segments are shown above and a vertical arrow at the 3'-end of the intI1 gene indicates the location of 108 bp deletion in AbaR3-type islands. Structures are drawn to scale for GenBank accession number KF483599.

B. Structures of Tn*6022* and Tn*6022*::Tn*2006* (AbaR4). Arrows indicate the orientation and extent of the genes and open reading frames and the antibiotic resistance gene is red. The position of Tn*2006*, which includes *oxa23* and two copies of ISAba1, is shown above. Structures are drawn to scale for GenBank accession number JN107991. Further details of these structures can be found in PMID: 20375036, PMID: 21873287 and PMID: 24080502.

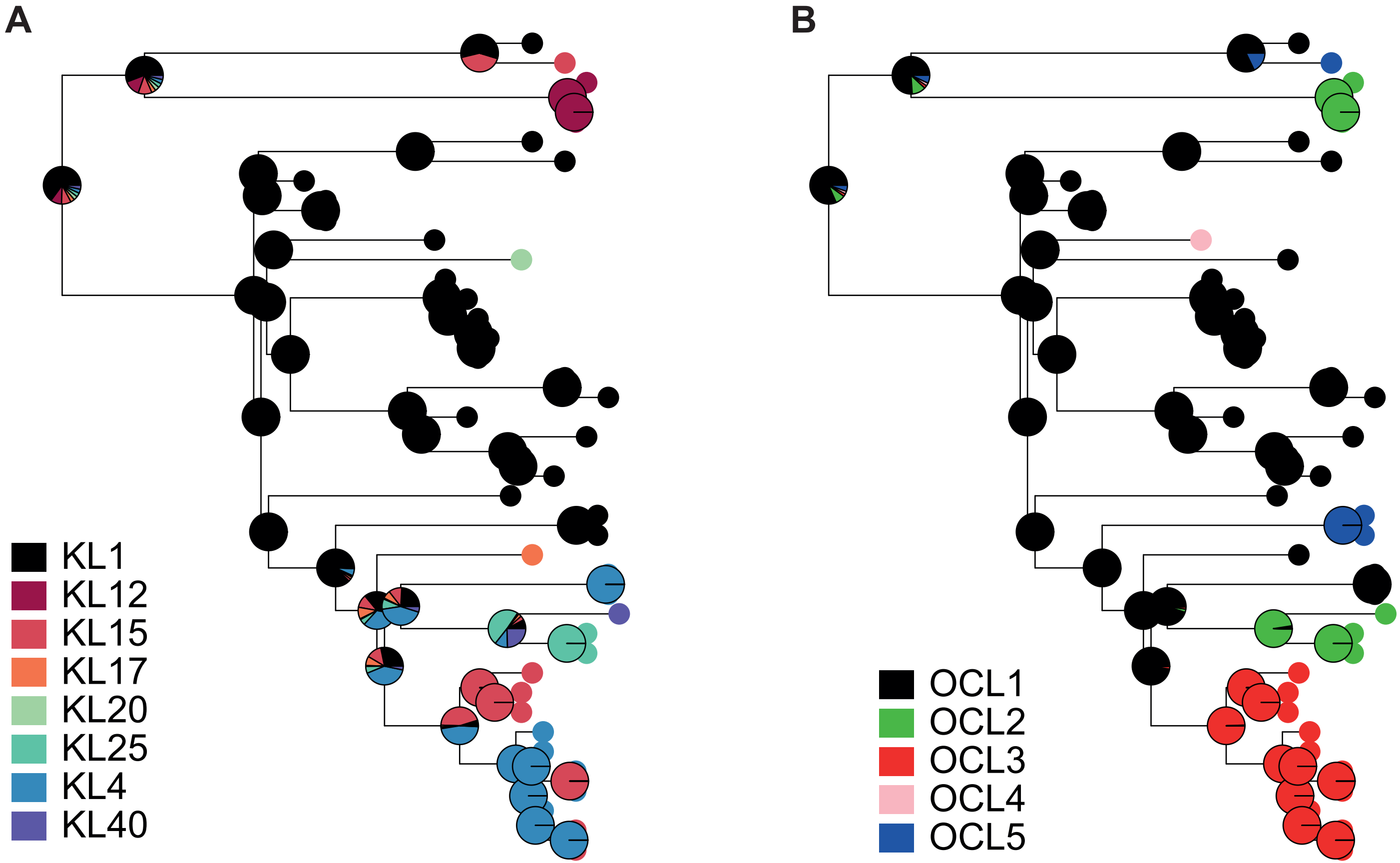

Figure S4. Maximum likelihood ancestral trait reconstruction for K and OC types. Pie graphs indicate the relative probability estimates of each K or OC locus occurring at each internal node in the tree.

Supplementary Table 1. Isolates sequenced and/or phenotyped for this study (continued on next page)

Strain[a]	Location of isolation	Year of isolation	Source	MLST (Institut Pasteur)	MLST [b] (Oxford)	Capsule locus	OC locus	*carO* allele [c]	Antibiotic resistance profile [d]
A1	UK	1982	nr [e]	ST1	ST231 (ST109)	KL1	OCL1	III	ApSmSpSuTcTp
A297 [g]	Netherlands	1984	nr	ST1	ST231 (ST109)	KL1	OCL1	III	ApSmSuTcTpKmTm
A388	Greece	2002	nr	ST1	ST439 (ST248)	KL20	OCL4	I	ApCTX,CAZ,TIM,IPM,MER,SmSpSuTcTpKmNmGmNeAkTm
J1	Sydney	1995	nr	ST1	ST231	KL1	OCL1	III	ApSmSpSuTcTpKmNmGmCIP,Nx
D3208	Sydney	1997	Blood	ST1	ST231 (ST109)	KL1	OCL1	III	ApSmSpSuTcTpKmNmGmCIP,Nx
J5	Sydney	1997	nr	ST1	ST231	KL1	OCL1	III	ApSmSpSuTcTpKmNmGmCIP,Nx
WM98	Sydney	1998	Wound	ST1	ST231 (ST109)	KL1a	OCL1	III	ApSmSpSuTcTpKmNmGmCIP,Nx
J7	Sydney	1998	nr	ST1	ST231	KL1a	OCL1	III	ApSmSpSuTcTpKmNmGmCIP,Nx
J10	Sydney	1999	nr	ST1	ST231	KL1a	OCL1	III	ApSmSpSuTcTpKmNmGmCIP,Nx
D2	Sydney	2006	Wound	ST1	ST231 (ST109)	KL1b	OCL1	III	ApSmSpSuTpKmNmGmCIP,Nx
D62	Sydney	2010	Midstream Urine	ST1	ST231 (ST109)	KL1b	OCL1	III	SpSuKmGmCIP,Nx
D30	Sydney	2008	Wound	ST1	ST231 (ST109)	KL1	OCL1	III	ApSmSpSuTpKmNmGmCIP,Nx
A83	Sydney	2002	-	ST1	ST231 (ST109)	KL1	OCL1	III	ApSmSpSuTpKmNmGmCIP,Nx
A92	Sydney	2005	Urine	ST1	ST231 (ST109)	KL1	OCL1	III	ApSmSpSuTpKmNmGmCIP,Nx
A85	Sydney	2003	Sputum	ST1	ST781 (ST126)	KL15	OCL3	III	ApCTX,CAZ,TIM,IPM,MER,SmSpSuTcTpKmNmGmCIP,Nx
6772166	Adelaide	2002	Pus	ST1	ST781 (ST126)	KL15	OCL3	III	ApCTX,CAZ,TIM,SmSpSuTcTpKmNmGmCIP,Nx
RBH3	Brisbane	2002	Endotracheal Aspirate	ST1	ST781 (ST126)	KL15	OCL3	III	ApCTX,CAZ,TIM,SmSpSuTcKmNmGmCIP,Nx
D15	Sydney	2009	Urine	ST1	ST231 (ST109)	KL1	OCL5	IV	ApCTX,CAZ,SmSpSuTpKmNmGmCIP,Nx
D13	Sydney	2009	Urine	ST1	ST231 (ST109)	KL1	OCL5	IV	ApCTX,CAZ,SmSpSuTpKmNmGmCIP,Nx
G7	Melbourne	2003	nr	ST1	ST231 (ST109)	KL17	OCL1	III	ApCTX,CAZ,TIM,SmSpSuTcTpKmNmGmAk
D81	Sydney	2010	Wound	ST1	ST441 (ST347)	KL4	OCL1b	III	ApCTX,CAZ,TIM,IPM,MER,SmSpSuTcKmNmGmAkCIP,Nx
D78	Sydney	2010	nr	ST1	ST441 (ST347)	KL4	OCL1b	III	ApCTX,CAZ,TIM,IPM,MER,SmSpSuTcKmNmGmAkCIP,Nx
D36	Sydney	2008	Wound	ST81	ST498 (ST247)	KL12	OCL2	III	ApCTX,CAZ,TIM,IMP,MER,SmSuTpKmGmTmCIP,Nx
AYE	France	2001	Pneumonia and UTI	ST1	ST231	KL1	OCL1	III	ApCTX,CAZ,SmSpSuTcTpKmNmGmNeTmCIP,Nx [i]
AB0057	USA	2004	Blood	ST1	ST207	KL4	OCL3	III	ApCTX,CAZ,TIM,IPM,MER,SmSpSuTcTpKmNmGmCIP,Nx [j]
AB5075	USA	2008	Bone infection	ST1	ST468	KL25	OCL2	III	ApCTX,CAZ,TIM,IPM,MER,SmSpSuTpKmNmGmNeAkTmCIP,Nx [k]

[a] We thank Kevin Towner for A1, A297 (RUH875) and A388 (10); Jon Iredell for isolates prefexed J or WM, which were reported in (22); other Australian isolates have been reported previously (5, 6, 7, 8, 9, 18, 19, 20 and 21). Complete genomes are available for AYE (GenBank accession number CU459141) (3), AB0057 (GenBank accession number CP001182) (2) and AB5075 (GenBank accession number CP008706) (4) and A1 (GenBank accession number CP010781) (11).

[b] The Oxford ST were determined from whole genome sequences. Oxford MLST scheme numbers in brackets indicate the ST determined by PCR and sequencing the products found in the references (5, 6, 7, 8 and 9).

[c] The *carO* variants have been previously classified in reference (17).

[d] Ap, ampicillin; CTX, cefotaxime; CAZ, ceftazidime; TIM, timentin (ticarcillin clavulanic acid); IPM, imipenem; MER, meropenem; Sm, streptomycin; Sp, spectinomycin; Su, sulphonamides; Tc, tetracycline; Tp, trimethoprim; Cl, chloramphenicol; Fl, florfenicol; Km, kanamycin; Nm, neomycin; Gm, gentamicin; Ne, netilmicin; Ak, amikacin; Tm, tobramycin; CIP, ciprofloxacin; Nx, nalidixic acid. Resistance profiles of the strains colored gray have been examined in the references (1) and (2) using a different antibiotic set.

[e] Not recorded.

[f] The annotated sequence of AbaR24 is in JN968482 and in the complete genome of A1(GenBank accession number CP010781).

[g] Also known as RUH875, the GC1 reference strain.

[h] AbaR4 is not a close relative of other AbaR types listed and is not derived from AbaR0. Hence, AbaR4 has been acquired independently.

[i] Antibiotic resistance profile determined in this study. AYE has also been reported as resistant to beta-lactams (except imipenem, piperacillin-tazobactam, and ticarcillinclavulanate), aminoglycosides, fluoroquinolones, chloramphenicol, tetracycline, and rifampin in reference (3).

[j] Antibiotic resistance profile determined in this study. AB0057 has also been reported as cefepime resistant in reference (1).

[k] In reference (12), AB5075 is reported as resistant to ampicillin/sulbactam, aztreonam, cefepime, ceftazidime, ciprofloxacin, gentamicin, imipenem, levofloxacin, and tobramycin and sensitive to tetracycline.

[l] Not applicable. AB5075 only includes, Tn*6019*::Tn*6018*, the Tn*6019* backbone transposon containing one copy of Tn*6018* (4). Tn*6019* is incorrectly annotated in (4).

[*] Public genome assembly only; all other assemblies were generated for this study.

References

1 **Adams, M. D., E. R. Chan, N. D. Molyneaux, and R. A. Bonomo.** 2010. Genome-wide analysis of divergence of antibiotic resistance determinants in closely related isolates of *Acinetobacter baumannii*. Antimicrob Agents Chemother 54:3569-3577. PMID: 20530228

2 **Adams, M. D., K. Goglin, N. Molyneaux, K. M. Hujer, H. Lavender, J. J. Jamison, I. J. MacDonald, K. M. Martin, T. Russo, A. A. Campagnari, A. M. Hujer, R. A. Bonomo, and S. R. Gill.** 2008. Comparative genome sequence analysis of multidrug-resistant *Acinetobacter baumannii*. J Bacteriol 190:8053-8064. PMID: 18931120

3 **Fournier, P.-E., D. Vallenet, V. Barbe, S. Audic, H. Ogata, L. Poirel, H. Richet, C. Robert, S. Mangenot, C. Abergel, P. Nordmann, J. Weissenbach, D. Raoult, and J.-M. Claverie.** 2006. Comparative genomics of multidrug resistance in *Acinetobacter baumannii*. PLoS Genet. 2:e7. PMID: 16415984

4 **Gallagher, L.A., E. Ramage, E. J. Weiss, M. Radey, H. S. Hayden, K. G. Held, H. K. Huse, D. V. Zurawski, M. J. Brittnacher, C. Manoil.** 2015. Resources for genetic and genomic analysis of emerging pathogen *Acinetobacter baumannii*. J Bacteriol 197:2027-35. PMID: 25845845

5 **Hamidian, M., and R. M. Hall.** 2011. AbaR4 replaces AbaR3 in a carbapenem-resistant *Acinetobacter baumannii* isolate belonging to global clone 1 from an Australian hospital. J Antimicrob Chemother 66:2484-2491. PMID: 21873287

6 **Hamidian, M., and R. M. Hall.** 2014. Tn*6168*, a transposon carrying an ISAba1-activated *ampC* gene and conferring cephalosporin resistance in *Acinetobacter baumannii*. J Antimicrob Chemother 69:77-80. PMID: 23920428

7 **Hamidian, M., K. E. Holt, D. Pickard, G. Dougan, and R. M. Hall.** 2014. A GC1 *Acinetobacter baumannii* isolate carrying AbaR3 and the aminoglycoside resistance transposon Tn*aphA6* in a conjugative plasmid. J Antimicrob Chemother 69:955-958. PMID: 24235096

8 **Hamidian, M., J. J. Kenyon, K. E. Holt, D. Pickard, and R. M. Hall.** 2014. A conjugative plasmid carrying the carbapenem resistance gene $bla_{\text{OXA-23}}$ in AbaR4 in an extensively resistant GC1 *Acinetobacter baumannii* isolate. J Antimicrob Chemother 69:2625-2628. PMID: 24907141

9 **Hamidian, M., M. Wynn, K. E. Holt, D. Pickard, G. Dougan, and R. M. Hall.** 2014. Identification of a marker for two lineages within the GC1 clone of *Acinetobacter baumannii*. J Antimicrob Chemother 69:557-558. PMID: 24080502

10 **Hamouda, A., B. A. Evans, K. J. Towner, and S. G. Amyes.** 2010. Characterization of epidemiologically unrelated *Acinetobacter baumannii* isolates from four continents by use of multilocus sequence typing, pulsed-field gel electrophoresis, and sequence-based typing of bla(OXA-51-like) genes. J Clin Microbiol 48:2476-2483. PMID: 20421437

11 **Holt, K.E., M. Hamidian, J. J. Kenyon, M. T. Wynn, J. Hawkey, D. Pickard, R. M. Hall.** 2015. Genome Sequence of *Acinetobacter baumannii* Strain A1, an Early Example of Antibiotic-Resistant Global Clone 1. Genome. Announc. 12;3. pii: e00032-15. doi: 10.1128/genomeA.00032-15. PMID: 25767221

12 **Jacobs, A. C., M. G. Thompson, C. C. Black, J. L. Kessler, L. P. Clark, C. N. McQueary, H. Y. Gancz, B. W. Corey, J. K. Moon, Y. Si, M. T. Owen, J. D. Hallock, Y. I. Kwak, A. Summers, C. Z. Li, D. A. Rasko, W. F. Penwell, C. L. Honnold, M. C. Wise, P. E. Waterman, E. P. Lesho, R. L. Stewart, L. A. Actis, T. J. Palys, D. W. Craft, and D. V. Zurawski.** 2014. AB5075, a Highly Virulent Isolate of Acinetobacter

13 **Kenyon, J. J., R. M. Hall.** 2013. Variation in the complex carbohydrate biosynthesis loci of *Acinetobacter baumannii* genomes. PLoS One 16;8:e62160. PMID: 23614028

14 **Kenyon, J. J., K. E. Holt, D. Pickard, G. Dougan, and R. M. Hall.** 2014. Insertions in the OCL1 locus of *Acinetobacter baumannii* lead to shortened lipooligosaccharides. Res Microbiol 165:472-5. PMID: 24861001

15 **Kenyon, J. J., A. M. Marzaioli, C. De Castro, and R. M. Hall.** 2015. 5,7-di-N-acetyl-acinetaminic acid: A novel non-2-ulosonic acid found in the capsule of an *Acinetobacter baumannii* isolate. Glycobiology 25: 644-654. PMID: 25595948

16 **Kenyon, J. J., S. J. Nigro, and R. M. Hall. 2014.** Variation in the OC locus of *Acinetobacter baumannii* genomes predicts extensive structural diversity in the lipooligosaccharide. PLoS One 23;9:e107833. PMID: 25247305

17 **Mussi, M. A., A. S. Limansky, V. Relling, P. Ravasi, A. Arakaki, L. A. Actis, and A. M. Viale.** 2011. Horizontal gene transfer and assortative recombination within the *Acinetobacter baumannii* clinical population provide genetic diversity at the single *carO* gene, encoding a major outer membrane protein channel. J Bacteriol 193:4736-4748. PMID: 21764928

18 **Nigro, S. J., V. Post, and R. M. Hall.** 2011. The multiresistant *Acinetobacter baumannii* European clone I type strain RUH875 (A297) carries a genomic antibiotic resistance island AbaR21, plasmid pRAY and a cluster containing ISAba1-*sul2*-CR2-*strB*-*strA*. J Antimicrob Chemother 66:1928-1930. PMID: 21642651

19 **Post, V., and R. M. Hall.** 2009. AbaR5, a large multiple-antibiotic resistance region found in *Acinetobacter baumannii*. Antimicrob. Agents Chemother 53:2667-2671. PMID: 19364869

20 **Post, V., M. Hamidian, and R. M. Hall.** 2012. Antibiotic-resistant *Acinetobacter baumannii* variants belonging to global clone 1. J Antimicrob Chemother 67:1039-1040. PMID: 22279182

21 **Post, V., P. A. White, and R. M. Hall.** 2010. Evolution of AbaR-type genomic resistance islands in multiply antibiotic-resistant *Acinetobacter baumannii*. J Antimicrob Chemother 65:1162-1170. PMID: 20375036

22 **Valenzuela, J. K., L. Thomas, S. R. Partridge, T. Reijden, L. Dijkshoorn, J. Iredell.** 2007. Horizontal gene transfer in a polyclonal outbreak of carbapenem-resistant *Acinetobacter baumannii*. J Clin Microbiol 45:453-60. PMID: 17108068

Supplementary Table 1. Isolates sequenced and/or phenotyped for this study (continued from previous page)

Strain[a]	AbaR type	AbaR accession number	AbaR Reference	KL and OCL accession number	KL and OCL reference	Accession number (reads)	No. reads	Mean read depth (×)	Accesion number (assembly)	Assembly size (bp)	No. contigs	N50 (bp)
A1	24	JN968482[f]	11	CP010781	13	ERR110077	305,288	63	CP010781	3,909,008	1 (Finished using PacBio)	
A297[g]	21	KM921776	18	-	-	ERR110085	305,288	98	FBWR01000000	4,113,004	105	418,925
A388	28	-	-	JQ684178	16 and this study	ERR110081	389,280	75	FBXE01000000	3,925,992	138	150,382
J1	0	-	-	-	-	ERR249288	515,971	95	FBWQ01000000	3,965,235	66	462,701
D3208	5	FJ172370	19	FJ172370	-	ERR119671	824,080	164	FBWZ01000000	3,940,429	89	354,592
J5	0	-	-	-	-	ERR249292	397,079	64	FBWP01000000	3,969,566	67	467,836
WM98	0	KF483599	9	KF483599	this study	ERR119657	1,266,600	247	FBXB01000000	4,069,158	81	358,980
J7	0	-	-	-	-	ERR249294	484,631	84	FBWT01000000	4,071,430	76	462,414
J10	0	-	-	-	-	ERR249297	678,612	115	FBWS01000000	4,080,334	77	462,517
D2	6	GQ406245	21	GQ406245	this study	ERR119664	970,762	190	FBWY01000000	3,956,778	96	218,350
D62	6	-	-	-	-	ERR119670	958,712	192	FBWW01000000	3,798,756	92	212,067
D30	26	KC665626	-	-	-	ERR110067	438,337	88	FBXG01000000	3,880,575	65	354,583
A83	7	-	-	-	-	ERR110098	377,590	78	FBWU01000000	3,858,636	53	358,985
A92	7	GQ406246	21	-	-	ERR110100	535,602	109	FBWV01000000	3,900,058	57	358,953
A85	3	KC118540	6, 8	KC118540	8, 13	ERR110086	305,379	60	FBXA01000000	4,083,615	124	292,020
6772166	3	-	6	-	-	ERR110082	454,791	73	FBWX01000000	4,005,394	111	351,586
RBH3	3	-	6	-	-	ERR119660	1,062,070	204	FBXD01000000	4,011,404	132	303,138
D15	8	-	-	-	-	ERR263719	4,861,924	110	FBXJ01000000	4,050,321	111	348,084
D13	8	HM590877	20	HM590877	16	ERR119668	1,221,556	234	FBXI01000000	4,049,172	112	267,583
G7	3	-	7	KC118541	this study	ERR110102	379,672	70	FBXF01000000	4,072,474	109	309,831
D81	23	JN409449	7	JN409449	13, 14	ERR119659	971,121	185	FBXC01000000	4,011,114	106	198,767
D78	23	-	7	-	-	ERR119679	957,401	185	FBXH01000000	4,010,315	105	198,760
D36	4[h]	JN107991	5	JN107991	13, 15	ERR119658	1,010,380	185	CP012952	4,147,982	1 (Finished using PacBio)	
AYE	1	CT025832	3	-	-	-	-	-	CU459141*	3,936,291	1 (Public finished genome)	
AB0057	3	CP001182	2	-	-	SRR1997868	4,869,486	134	CP001182*	4,050,513	1 (Public finished genome)	
AB5075	na[l]	CP008706	4	BK008886	this study	-	-	-	AHAH00000000*	3,984,833	99	136,399

Table S2. Public genome data used in this study.

Strain	Location of isolation	Year of isolation	Source	MLST (Pasteur) [a]	MLST (Oxford) [a]	Capsule locus	OC locus	*carO* allele [b]	AbaR type	Accession number (assembly)	Accession number (reads)	Reference
TG19582	nr [c]	nr	nr	ST1	ST231	KL1	OCL1	III	No AbaR	AMIV00000000	-	5
307-0294 [d]	USA	1994	Blood	ST1	ST231	KL1	OCL1	III	No AbaR	CP001172	-	2
NIPH 527[e]	Netherlands	1984	Urine	ST1	ST231	KL1	OCL1	III	[21] [f]	APQW00000000	SRR654309	6
NIPH 290	Netherlands	1994	Urine	ST1	ST231	KL1	OCL1	III [g]	0	APRD00000000	SRR654194	6
OIFC074	USA	2003	nr	ST19	ST231	KL1	OCL5	II	Tn*6022*	AMDE00000000	SRR387244	3
AB058[h]	USA	2003	Blood	ST20	ST449	KL1[i]	OCL1[i]	II	10	ADHA00000000	-	1
AB056[j]	USA	2004	Blood	ST1	ST207	KL4[i]	OCL3[i]	III	9	ADGZ00000000	-	1
AB059[k]	USA	2004	Blood	ST1	ST207	KL4[i]	OCL3[i]	III	3	ADHB00000000	-	1
Naval-21	USA	2006	Wound	ST19	ST946	KL15	OCL1	II	Tn*6022*	AMSY00000000	SRR387323	3
Naval-83	USA	2006	Wound	ST20	ST449	KL1c	OCL1	II	10	AMFK00000000	SRR387319	3
6013113	UK	2007	Skin	ST81	ST372	KL12	OCL2[i]	III	No AbaR	ACYR00000000	SRR089343	-
6013150	UK	2007	Skin	ST81	ST498	KL12	OCL2	III	No AbaR	ACYQ00000000	SRR089344	-
908-13	USA	2007	Urine	ST1	ST207	KL4	OCL3	III	Tn*6019-6018*	AMHW00000000	-	5
909-02-7	USA	2007	Sputum	ST1	ST207	KL4[i]	OCL3	III	Tn*6019-6018*	AMHZ00000000	-	5
Canada-BC1	Canada	2007	nr	ST1	ST947	KL15	OCL3	III	[29] [l]	AMSZ00000000	SRR387315	3
Canada-BC5	Canada	2007	nr	ST1	ST947	KL15	OCL3	III	29	AFDN00000000	SRR353953	3
IS-58	nr	2008	Respiratory tract	ST1	ST945	KL25	OCL2	III	10	AMGH00000000	SRR387296	3
ANC 4097	nr	2011	Tracheal aspirate	ST1	ST441	KL40	OCL2	III	3	APRF00000000	SRR654201	-

[a] IP; Institut Pasteur scheme, O; Oxford scheme.

[b] The *carO* variants have been previously described in reference (4)

[c] not recorded.

[d] AB307-0294 is an antibiotic sensitive strain.

[e] also known as RUH875 (5).

[f] bracket indicates that NIPH 527 is likely to contain AbaR21.

[g] interrupted by ISAba125

[h] AB058 is recorded as ceftazidime, cefepime, amikacin, tobramycin and ciproflixacin resistant in reference (1).

[i] these loci are not in a single contig and may include IS.

[j] AB056 is recorded as ampicillin/sulbactam, ceftazidime, cefepime, imipenem, meropenem and ciproflixacin resistant in reference (1)

[k] AB059 is recorded as ampicillin/sulbactam, ceftazidime, cefepime, imipenem, meropenem, amikacin and ciproflixacin resistant in reference (1)

[l] likely to contain AbaR29.

References

1. Adams, M. D., E. R. Chan, N. D. Molyneaux, and R. A. Bonomo. 2010. Genome-wide analysis of divergence of antibiotic resistance determinants in closely related isolates of *Acinetobacter baumannii*. Antimicrob Agents Chemother 54:3569-3577. PMID: 20530228

2. Adams, M. D., K. Goglin, N. Molyneaux, K. M. Hujer, H. Lavender, J. J. Jamison, I. J. MacDonald, K. M. Martin, T. Russo, A. A. Campagnari, A. M. Hujer, R. A. Bonomo, and S. R. Gill. 2008. Comparative genome sequence analysis of multidrug-resistant *Acinetobacter baumannii.* J Bacteriol 190:8053-8064. PMID: 18931120

3. Chan AP, Sutton G, DePew J, Krishnakumar R, Choi Y, Huang XZ, Beck E, Harkins DM, Kim M, Lesho EP, Nikolich MP, Fouts DE. A novel method of consensus pan-chromosome assembly and large-scale comparative analysis reveal the highly flexible pan-genome of *Acinetobacter baumannii.* Genome Biol 2015 Jul 21;16:143. doi: 10.1186/s13059-015-0701-6. PMID: 26195261

4. Mussi, M. A., A. S. Limansky, V. Relling, P. Ravasi, A. Arakaki, L. A. Actis, and A. M. Viale. 2011. Horizontal gene transfer and assortative recombination within the *Acinetobacter baumannii* clinical population provide genetic diversity at the single *carO* gene, encoding a major outer membrane protein channel. J bacteriol 193:4736-4748. PMID: 21764928

5. Sahl, J.W., J. D. Gillece, J. M. Schupp, V. G. Waddell, E. M. Driebe, D. M. Engelthaler, P. Keim. 2013. Evolution of a pathogen: a comparative genomics analysis identifies a genetic pathway to pathogenesis in *Acinetobacter.* PLoS One 8:e54287. doi: 10.1371/journal.pone.0054287. PMID: 23365658

6. Touchon, M., J. Cury, E. J. Yoon, L. Krizova, G. C. Cerqueira, C. Murphy, M. Feldgarden, J. Wortman, D. Clermont, T. Lambert, C. Grillot-Courvalin, A. Nemec, P. Courvalin, and E. P. Rocha. 2014. The genomic diversification of the whole *Acinetobacter* genus: origins, mechanisms, and consequences. Genome Biol Evol 6:2866-2882. PMID: 25313016

Table S3. Spatially clustered codons (separated by ≤5 codons) that harbour multiple independent substition mutations within the GC1 population. Key for note column: <-> indicates that both the indicated substitution and its reversion were detected in different parts of the GC1 tree; (xN) indicates that the indicated substitution mutation was detected multiple times, in N different branches of the GC1 tree. * indicates codons affected by multiple independent mutations (details shown in Table 1). Genes related to antimicrobial resistance are highlighted in bold. ***(continued next page)***

Gene	Product	Codon No.	Codon Change	Amino Acid Change	Type	A1 coordinate	Note
ABA1_00068	Putative aliphatic sulfonates-binding protein precursor	212*	ACA->ACG	T->T	s	68789	(<->)
ABA1_00088	Wzc, involved in capsule export	545	GGT->TGT	G->C	ns	88705	
		547	TCG->ACG	S->T	ns	88699	
ABA1_00091	UDP-N-acetyl-galactosamine dehydrogenase Gna	30	GTA->GTT	V->V	s	92338	
		31	GTC->GTT	V->V	s	92341	
		32*	GGA->GGC	G->G	s	92344	
		32	GGT->GGC	G->G	s	92344	
		35	ATC->ATT	I->I	s	92353	
ABA1_00155	BolA family transcriptional regulator	91*	TCA->CCA	S->P	ns	171998	(<->)
ABA1_00215	hypothetical protein	39	GAC->GAG	D->E	ns	226421	
		41	CTC->TTC	L->F	ns	226425	
ABA1_00226	**ParC DNA topoisomerase IV, A subunit**	84*	TCG->TTG	S->L	ns	239386	(x5)
		84	TTG->TGG	L->W	ns	239386	
		88	GAA->AAA	E->K	ns	239397	
ABA1_00294	Sulphate permease, Sup	35	TCT->TCA	S->S	s	302471	
		40	GTT->GTA	V->V	s	302456	
ABA1_00716	hypothetical protein	377*	GGA->AGA	G->R	ns	757604	(x2)
ABA1_00943	Protein MosA	298	GCA->GAA	A->E	ns	994836	
		302	GCC->GCT	A->A	s	994823	
ABA1_01174	hypothetical protein	34	GTT->GCT	V->A	ns	1227313	
		36	GAA->GTA	E->V	ns	1227319	
ABA1_01266	AhpF alkyl hydroperoxide reductase, F subunit	433*	GCG->ACG	A->T	ns	1332044	
		433	GCG->GCA	A->A	s	1332042	
ABA1_01701	Cupin superfamily protein	163	GTA->GCA	V->A	ns	1781284	
		166	CAA->AAA	Q->K	ns	1781276	
ABA1_01702	Septum formation protein Maf	179	GGT->GGG	G->G	s	1781859	
		181	CCC->TCC	P->S	ns	1781855	
ABA1_01794	PepSY-associated TM helix family protein	138	TTC->ATC	F->I	ns	1878415	
		141	TTA->TCA	L->S	ns	1878425	
ABA1_01811	hypothetical protein	93	CCT->CCA	P->P	s	1897193	
		94	GAA->GGA	E->G	ns	1897191	
ABA1_01827	Ribonuclease D (RNase D)	152	AAT->AAA	N->K	ns	1908931	
		154	GTT->ATT	V->I	ns	1908935	
ABA1_01844	Dihydrolipoyllysine-residue acetyltransferase component of pyruvatedehydrogenase complex(E2)	296	GCG->GTG	A->V	ns	1925851	
		298	GCT->GCC	A->A	s	1925858	
ABA1_01920	F17 fimbrial protein precursor	133	CAA->CGA	Q->R	ns	2009574	
		137	CAA->AAA	Q->K	ns	2009563	
ABA1_01929	**AcrB protein**	87	GGT->AGT	G->S	ns	2023580	
		90	GAG->GTG	E->V	ns	2023570	
		97*	CCG->CTG	P->L	ns	2023549	(x2)
ABA1_01931	**Two-component response regulator AdeR**	24	ATT->ACT	I->T	ns	2025241	
		26*	GAC->AAC	D->N	ns	2025246	
		26	GAC->GTC	D->V	ns	2025247	
ABA1_01932	**Two-component response regulator AdeS**	51	GAA->AAA	E->K	ns	2026096	
		55	AGT->AAT	S->N	ns	2026109	
		94	GTC->GCC	V->A	ns	2026226	
		97	GCA->ACA	A->T	ns	2026234	
ABA1_01953	**MFS transporter, drug:H+ antiporter-1**	25*	TTT->ATT	F->I	ns	2050142	
		25	TTT->CTT	F->L	ns	2050142	
		327	CCG->TCG	P->S	ns	2049236	
		328	TTC->TCC	F->S	ns	2049232	
ABA1_02065	PcaH protocatechuate 3,4-dioxygenase, beta subunit	144*	AAA->ACA	K->T	ns	2166536	(x2)
ABA1_02134	Diguanylate cyclase (GGDEF) domain protein	579	CTA->CTG	L->L	s	2237948	
		580	AAA->ACA	K->T	ns	2237950	
ABA1_02141	Proline-specific permease proY	233*	CCG->CCA	P->P	s	2246974	(x2)
ABA1_02240	FhuE receptor precursor	263	GAT->GTT	D->V	ns	2350844	
		267	GGA->GGG	G->G	s	2350831	
ABA1_02258	Protein U precursor	181	TGT->TAT	C->Y	ns	2366783	
		183	GAT->GTT	D->V	ns	2366777	
ABA1_02377	Cyclopropane-fatty-acyl-phospholipid synthase family protein	62	AAT->AAA	N->K	ns	2501896	
		65	TTG->TAG	L->*	ns	2501888	
ABA1_02431	Serine/threonine-protein kinase 1	55*	TTT->TAT	F->Y	ns	2550200	(x2)
ABA1_02631	**Beta-lactamase class C, ampC**	341*	AAC->ACC	N->T	ns	2734409	(x2)

Table S3. Spatially clustered codons (separated by ≤5 codons) that harbour multiple independent substition mutations within the GC1 population. *(continued from prev page)*

Gene	Product	Codon No.	Codon Change	Amino Acid Change	Type	A1 coordinate	Note
ABA1_02648	Ferric anguibactin receptor precursor (OM2)	392*	GCT->ACT	A->T	ns	2756443	(x3)
		392	GCT->GAT	A->D	ns	2756442	
		392	GCT->GTT	A->V	ns	2756442	
ABA1_02860	**DNA gyrase, A subunit GyrA**	81*	TCA->TTA	S->L	ns	2992514	(X4)
ABA1_02933	hypothetical protein	391	ACA->AGA	T->R	ns	3078412	
		395	ATG->ATT	M->I	ns	3078399	
ABA1_03474	**Penicillin binding protein**	512	GCA->TCA	A->S	ns	3625121	
		515	GCC->GTC	A->V	ns	3625111	
ABA1_03676	Proline-specific permease proY	331*	AGT->GGT	S->G	ns	3840150	(<->)
ABA1_03708	DNA-3-methyladenine glycosylase 1	24*	GGC->GGT	G->G	s	3871606	(<->)

Table S4. Results of BEAST analyses. Results are shown for combinations of two clock models (strict or relaxed clock), including or excluding the 454 sequenced genome 307-0294. As explained in the text, the final results reported in the text and shown in Figure 3 and Figure S2 are those using a strict clock without 307-0294 (part **A**).
(continued on next page)

(A) Strict Clock, without 454 - RESULTS REPORTED IN TEXT

Summary Statistic	likelihood	treeModel.rootHeight	tMRCA(Lineage 1)	tMRCA(Lineage 2)	clock.rate
mean	-12239.7598	51.2726	33.6429	43.6976	2.46E-03
stderr of mean	0.0438	0.0483	0.0229	0.0423	2.68E-06
stdev	5.6221	2.6757	0.9316	3.072	1.29E-04
variance	31.6082	7.1594	0.8678	9.4375	1.65E-08
median	-12239.4198	51.1631	33.5813	43.5688	2.46E-03
mode	n/a	n/a	n/a	n/a	n/a
geometric mean	n/a	51.2032	33.63	43.5903	2.46E-03
95% HPD Interval	[-12250.9303, -12229.1215]	[46.0987, 56.5585]	[31.8712, 35.4911]	[37.8147, 49.7679]	[2.2132E-3, 2.7178E-3]
auto-correlation time (ACT)	3941.4258	21084.1637	43.5989	13.6679	28142.2278
effective sample size (ESS)	16442.7807	3073.7762	1651.6016	5268.4152	2302.8738

(B) Strict Clock, with 454

Summary Statistic	likelihood	treeModel.rootHeight	tMRCA(Lineage 1)	tMRCA(Lineage 2)	clock.rate
mean	-12715.3818	48.6972	32.9567	44.0586	2.36E-03
stderr of mean	0.0371	0.0408	0.0193	0.0406	2.30E-06
stdev	5.4645	2.3956	0.8154	3.1071	1.18E-04
variance	29.8611	5.7388	0.6648	9.6543	1.38E-08
median	-12715.0149	48.5785	32.8896	43.8966	2.36E-03
mode	n/a	n/a	n/a	n/a	n/a
geometric mean	n/a	48.6386	32.9467	43.95	2.36E-03
95% HPD Interval	[-12726.4007, -12705.2781]	[44.0675, 53.4197]	[31.4415, 34.5816]	[38.0465, 50.2409]	[2.1328E-3, 2.5944E-3]
auto-correlation time (ACT)	2987.4109	18795.7594	40.1689	12.314	24781.6867
effective sample size (ESS)	21693.7011	3448.0118	1792.6322	5847.6379	2615.1569

(C) Relaxed Clock, without 454

Summary Statistic	likelihood	treeModel.rootHeight	tMRCA(Lineage 1)	tMRCA(Lineage 2)	meanRate	ucld.mean	ucld.stdev	coefficientOf Variation
mean	-12186.5041	61.0668	35.2964	50.9753	2.43E-03	2.50E-03	0.605	0.6036
stderr of mean	0.037	0.3335	0.063	0.47	9.81E-06	8.45E-06	1.98E-03	2.11E-03
stdev	7.5127	16.9635	3.66	21.1756	3.92E-04	4.38E-04	0.1436	0.1458
variance	56.4399	287.7591	13.3954	448.4081	1.53E-07	1.92E-07	0.0206	0.0213
median	-12186.1492	58.5673	34.5701	49.2148	2.40E-03	2.45E-03	0.5897	0.5847
mode	n/a	n/a	n/a	n/a	n/a	n/a	n/a	n/a
geometric mean	n/a	58.9477	35.1389	46.6064	2.40E-03	2.47E-03	0.5885	0.587
95% HPD Interval	[-12201.2842, -12172.085]	[31.1084, 92.2934]	[30.534, 41.2165]	[14.91, 89.2336]	[1.6922E-3, 3.2388E-3]	[1.7351E-3, 3.3844E-3]	[0.3451, 0.8918]	[0.3524, 0.9]
auto-correlation time (ACT)	17956.5582	2.87E+05	24.4177	40.5861	4.65E+05	2.77E+05	1.40E+05	1.56E+05
effective sample size (ESS)	41300.7878	2586.445	3374.6811	2030.3026	1593.6184	2680.5509	5279.9546	4765.5798

(D) Relaxed Clock, with 454

Summary Statistic	likelihood	treeModel.rootHeight	tMRCA(Lineage 1)	tMRCA(Lineage 2)	meanRate	ucld.mean	ucld.stdev	coefficientOf Variation
mean	-12645.3175	54.0386	34.4145	41.761	2.53E-03	2.63E-03	0.6572	0.6557
stderr of mean	0.0377	0.4015	0.0742	0.6089	1.35E-05	1.20E-05	2.52E-03	2.75E-03
stdev	7.2723	16.0747	3.872	21.9491	4.56E-04	5.24E-04	0.1476	0.1542
variance	52.8856	258.3945	14.9926	481.7636	2.08E-07	2.75E-07	0.0218	0.0238
median	-12644.9705	51.0943	33.5729	36.1207	2.47E-03	2.54E-03	0.6415	0.6343
mode	n/a	n/a	n/a	n/a	n/a	n/a	n/a	n/a
geometric mean	n/a	51.9672	34.2434	36.6312	2.49E-03	2.58E-03	0.6411	0.6388
95% HPD Interval	[-12659.9532, -12631.5859]	[30.226, 83.8079]	[30.0161, 40.2496]	[13.0875, 83.0738]	[1.7173E-3, 3.4931E-3]	[1.7667E-3, 3.7056E-3]	[0.3889, 0.9533]	[0.3837, 0.958]
auto-correlation time (ACT)	20074.726	4.67E+05	30.5822	64.0388	6.56E+05	3.93E+05	2.18E+05	2.39E+05
effective sample size (ESS)	37301.6299	1602.578	2720.6024	1299.2436	1142.1205	1905.6774	3433.4163	3133.8716

(E) Summary

Clock model	tMRCA(GC1)	tMRCA(Lineage 1)	tMRCA(Lineage 2)	rate
***Strict Clock, without 454**	[46.0987, 56.5585]	[31.8712, 35.4911]	[37.8147, 49.7679]	[2.2132E-3, 2.7178E-3]
**Strict Clock, with 454**	[44.0675, 53.4197]	[31.4415, 34.5816]	[38.0465, 50.2409]	[2.1328E-3, 2.5944E-3]
**Relaxed Clock, without 454**	[31.1084, 92.2934]	[30.534, 41.2165]	[14.91, 89.2336]	[1.6922E-3, 3.2388E-3]
**Relaxed Clock, with 454**	[30.226, 83.8079]	[30.0161, 40.2496]	[13.0875, 83.0738]	[1.7173E-3, 3.4931E-3]

Table S5. GenBank accession numbers for K gene clusters.

Name	GenBank accession number[a]	Base Coordinates
KL1	CP010781	87963-110544
KL1a	KF483599.2	724-24299
KL1b	GQ406245.5	724-25628
KL2	KJ459911.2	3057-27820
KL3	KF793926.1	1-27785
KL4	JN409449.3	1-33327
KL5	NC_010400[b]	58381 - 88436
KL6	KF130871.1	1-28301
KL7	NC_017387 [b]	77073 - 107448
KL8	CP003847 [b]	90803 - 127124
KL9	JN247441.4	1-30189
KL10	CP003856.1[b]	304605-332863
KL12	JN107991.2	3247-38792
KL13	AEPK01000012.1[b]	32538-70756
KL14	KF030679.2	724-21932
KL15	KC118540.6	9179-37114
KL17	KC118541.2	3247-25474
KL20	JQ684178.2	3250-32301
KL23	AFDL01000004.1[b]	695766-722540
KL25	BK008886	1-23498
KL27	KT266827.1	1-32668
KL28	AMTB01000027 [b]	32705 - 60750
KL30	APPL01000041.1[b]	82987- 106722
KL32	KT359615.1	1-24182
KL33	APRA01000009.1[b]	555847-581577
KL37	APOU01000009.1 [b]	32574-53092
KL40	KP100029.1	724-24731
KL42	APOF01000020.1[b]	103755-129611
KL44a	APRC01000043 [b]	97989 - 131451
KL45	APQV01000013.1 [b]	342271-366295
KL46	APQY01000009.1[b]	583068-609640
	APOV01000005.1 [b]	12859-36514
KL49	KT359616.1	1-34551
KL58	KT359617.1	1-27015
KL63	KC526909 [b,c]	1-30479
KL78	AVOE01000009.1 [b]	32365-69506
KL79	JEYA01000007.1 [b]	112492-145289
KL91	KM402814.1	169-26579

[a] Accession number of contig that includes the capsule gene cluster.
[b] Annotated differently to scheme reported in Kenyon and Hall, 2013
[c] Sequence including *fkpA-wzc-wzb-wza* is missing from GenBank entry
